# Early Differentiation of Barley Diseases (Net Blotch, Spot Blotch, and Stem Rust) Using Hyperspectral Imaging and Machine Learning

**DOI:** 10.3390/jof12100793

**Published:** 2026-10-21

**Authors:** Anton Terentev, Danila Eremenko, Ekaterina Shaydayuk, Daria Kuznetsova, Alexander Fedotov

**Affiliations:** 1 All-Russian Institute of Plant Protection, 196608 Saint Petersburg, Russia; 2 Institute of Cybersecurity and Computer Science, Peter the Great St. Petersburg Polytechnic University, 195251 Saint Petersburg, Russia

**Keywords:** barley, *Puccinia graminis*, *Pyrenophora teres*, *Bipolaris sorokiniana*, hyperspectral imaging, spectral features, early plant disease detection, machine learning, multiclass classification, ablation study

## Abstract

Early and accurate detection of fungal diseases is critical for sustainable barley production, yet multiclass differentiation at early stages remains a major challenge. This study evaluated whether hyperspectral imaging (450–874 nm) combined with machine learning could distinguish three barley pathogens—*Puccinia graminis* f. sp. *tritici* (stem rust), *Pyrenophora teres* (net blotch), and *Bipolaris sorokiniana* (spot blotch)—during the early phase. We used a systematic ablative approach, comparing three classifiers (Logistic Regression, PLS-DA, and kNN) and several spectral feature representations across eight experimental splits to assess cross-experimental generalization. The best configuration, PLS-DA with spline-based features, achieved a mean balanced accuracy of 0.803 across the eight test experiments, with a standard deviation of 0.076. Classification performance increased steadily over the observation period, reaching a plateau above 0.900 from the third imaging day onward. Stem rust was consistently more difficult to classify than both blotch diseases; the reason for this remains unclear, although a longer latent period is a plausible factor. The results demonstrate that multiclass spectral differentiation of these three barley diseases is feasible at early stages under rigorous cross-experimental validation.

## 1. Introduction

Barley (*Hordeum vulgare* L.) is one of the earliest domesticated and most widely adapted cereal crops and it is cultivated extensively worldwide. It consistently ranks among the top four cereals in production, trailing only wheat, maize, and rice, with approximately 60 million hectares of agricultural land dedicated to its cultivation and annual output reaching around 150 million tons [1]. While barley was initially cultivated for human consumption, the bulk of modern barley production (~70%) is now directed toward animal feed, with the remaining share consumed in malting, brewing, and food-processing industries [2].

Barley is affected by a wide range of fungal pathogens that can significantly reduce grain yield and overall crop productivity [3]. Among the most widespread and damaging diseases is net blotch, caused by *Pyrenophora teres* Drechs., which can cause substantial yield losses and, in severe cases, complete crop failure [4,5]. Spot blotch, associated with *Bipolaris sorokiniana* (Sacc.) Shoem., can reach epidemic proportions, impairing seed quality and reducing germination capacity [5,6]. Stem rust, caused by *Puccinia graminis* f. sp. *tritici*, also poses a threat to barley production, as new virulent races such as TTKSK (Ug99) may overcome resistance and cause large yield losses [7,8,9]. Collectively, these three pathogens represent major constraints to barley production in many growing regions worldwide.

Hyperspectral remote sensing is increasingly recognized as a promising tool for early, non-invasive detection of plant diseases, potentially enabling interventions before visible symptoms emerge [10,11,12]. Various studies have demonstrated the potential of hyperspectral sensing for detecting barley spot blotch [13,14], barley net blotch [13,15] and barley stem rust [13,16] including early detection. However, there are very few studies addressing the question of whether machine learning can distinguish different diseases at early stages with an accuracy significantly above random chance.

In a series of preceding studies, we have explored this potential, initially developing a systematic preprocessing pipeline and machine learning framework for the early detection of wheat brown rust on detached leaves [17] and later of wheat stem rust on intact plants under controlled conditions [18]. This approach, combining pixel-wise correction, curve-wise normalization, smoothing, and channel-wise standardization, was shown to be capable of addressing the task of detecting early infections. We subsequently investigated the transferability of these models between wheat and barley [19]. Furthermore, the original pipeline, optimized for disease diagnostics, was adapted and tested for a healthy plant phenotyping task involving wheat, barley, and rye [20]. Despite these advancements, none of the developed methods directly addressed the more complex challenge of early multiclass classification among different pathogens.

The three pathogens differ in their early infection strategies. *P. graminis* f. sp. *tritici* is an obligate biotroph that grows intercellularly but forms haustoria to penetrate host mesophyll cells. *B. sorokiniana* is a hemibiotroph that penetrates epidermal cells via appressoria, forms intracellular infection hyphae, and subsequently switches to necrotrophic growth in the mesophyll. *P. teres* f. *teres* is a hemibiotroph/necrotroph that rapidly colonizes the mesophyll, with a very short or absent biotrophic phase. Despite these biological differences, the early symptoms induced by these pathogens are qualitatively similar, making visual differentiation difficult [21,22,23,24]. This provides a plausible basis for spectral differentiation: the pathogens are biologically distinct from the earliest stages, yet this distinctness is not readily apparent to the naked eye. Consequently, the host’s physiological response such as localized suppression of photosynthesis, mild oxidative stress, and subtle changes in pigment composition is qualitatively similar regardless of which pathogen is present, making early differentiation fundamentally challenging [22,25]. Later the pathogens develop to the next phases and distinct symptoms such as lesions, chlorosis, or pustules emerge [21,22]. The timing of this transition is plastic and depends critically on environmental conditions (temperature, humidity, light intensity), inoculum density, and host susceptibility [22,26]. Under optimal laboratory conditions (high inoculum, controlled climate), this switch can occur as early as 2–3 DAI for *P. teres* and *B. sorokiniana*, with primary chlorosis potentially manifested and visible to the naked eye within 36–48 h already. Conversely, under suboptimal field conditions, the asymptomatic biotrophic phase can be dramatically prolonged, lasting up to 10–14 days or more, as hyphal growth slows considerably [26,27,28]. Consequently, while the order of symptom onset (*P. teres/B. sorokiniana* earlier than *P. graminis*) remains consistent, the absolute DAI (day after inoculation) values are highly context-dependent. This temporal plasticity does not invalidate the concept of an early disease detection window, but it underscores that the window’s duration is a variable, not a fixed parameter—making robust early detection even more challenging, yet also more practically relevant across diverse agronomic scenarios. However, the pathogens themselves are biochemically distinct, showing high similarity in their composition within a species despite their fungal origin, especially at the later development stages [29,30,31]. This specific biochemical composition suggests that, despite the similarity in the host’s generic stress response, there are intrinsic spectral differences between the pathogens themselves that a machine learning model could, in principle, learn to distinguish, although their specific connection with the spectra is still very unclear [32]. This premise underpins our proof-of-concept study: to determine whether such subtle, pathogen-specific spectral signatures can be extracted from data dominated by the host response. We recognize that laboratory-based experiments do not replicate the full complexity of field conditions, including variable lighting, plant phenology, and mixed infections. Nevertheless, a proof-of-concept demonstration in a controlled setting is a necessary prerequisite for eventual field deployment.

Therefore, the primary goal of this study was to determine whether hyperspectral imaging (450–874 nm) combined with machine learning can differentiate three barley pathogens (*Puccinia graminis* f. sp. *tritici*, *Pyrenophora teres*, and *Bipolaris sorokiniana*) during the early stages of infection under controlled conditions. Specifically, we aimed to (i) quantify the achievable multiclass accuracy under cross-experimental validation, and (ii) identify the most informative spectral feature representation and classifier for this task.

## 2. Materials and Methods

### 2.1. Plant Material and Inoculum Preparation

The isolate of *P. graminis* f. sp. *tritici* was collected by plant pathologists from the All-Russian Institute of Plant Protection (Saint Petersburg, Russia) from spring wheat crops in the Chelyabinsk region during July 2025 and was maintained in the live culture collection of the All-Russian Institute of Plant Protection (VIZR), Saint Petersburg, Russia. Species identity was confirmed morphologically by microscopic examination of the collected urediniospores. The forma specialis *tritici* was determined by host range, which is the standard method for this purpose. *P. graminis* f. sp. *secalis* and *P. graminis* f. sp. *avenae* do not infect wheat under field conditions. Infected plants were dried and kept as herbarium samples. The fungal population was initially propagated on the highly susceptible winter wheat cv. ‘Michigan Amber’ using 10-day-old intact wheat seedlings (first true leaf stage) according to established protocols [33]. To obtain barley-adapted races, urediniospores from this population were then used to inoculate barley cv. ‘Lyuboyar’. Urediniospores were collected from barley plants showing typical stem rust symptoms, and the population was confirmed to be *P. graminis* f. sp. *tritici* by microscopic examination of urediniospore morphology (Figure S1A). This population was subsequently used for all experiments in this study. Urediniospores were gently tapped into glass tubes and used immediately for plant inoculation. Spore count was performed using a C-Chip hemocytometer (NanoEntek, Seoul, Republic of Korea) and the concentration was adjusted to 10,000 urediniospores/mL [19].

The isolates of *P. teres* and *B. sorokiniana* were collected by plant pathologists from the Laboratory of Mycology and Phytopathology (All-Russian Institute of Plant Protection, Saint Petersburg, Russia) from commercial spring barley fields in the Leningrad region during July–August 2025 and were maintained in the live culture collection of the All-Russian Institute of Plant Protection (VIZR), Saint Petersburg, Russia. Species identity was confirmed morphologically by microscopic examination of conidial morphology (Figure S1B,C). The isolates were cultured on a modified Chapek medium composed of KCl—0.5 g, KH_2_PO_4_—0.5 g, MgSO_4_—0.5 g, urea—1.2 g, lactose—20 g, and agar—20 g per liter of distilled water. Incubation was performed at ambient temperature under artificial illumination with a photosynthetic photon flux density of 85 µmol m^−2^ s^−1^ (3000 lux) and a 16/8 h light/dark cycle for 10 days [34]. The *P. teres* and *B. sorokiniana* cultures were then flooded with distilled water supplemented with 0.01% TWEEN 20 (Sigma-Aldrich, St. Louis, MO, USA), and conidia were collected by adding sterile water to the Petri dishes and scraping them off with a sterile spatula. The resulting conidial suspension was filtered through gauze. Spore concentrations were adjusted to 5000 conidia/mL for *P. teres* [35] and 12,000 conidia/mL for *B. sorokiniana* [36] prior to inoculation.

The study utilized spring barley cv. ‘Lyuboyar’ susceptible to *P. graminis* f. sp. *tritici*, *P. teres* and *B. sorokiniana.* Seeds were obtained from the Branch of the Federal State Budgetary Institution “State Commission of the Russian Federation for Selection Achievements Test and Protection” for the Leningrad Region. In each experiment, barley was sown in 12 plastic containers (320 × 220 × 160 mm) with 70–75 seeds per container. Each experiment was repeated two times. Containers were divided into two equal groups (control and inoculated). Plants were maintained under optimal conditions (watering, fertilization) in a phytotron at 23–25 °C, with a 16 h photoperiod, photosynthetic photon flux density of 202.5–270 µmol m^−2^ s^−1^ (15,000–20,000 lux), and 60–70% humidity.

Plants were inoculated at growth stage 12 on the BBCH scale (two true leaves stage). Plants were sprayed with a spore suspension with NOVEC 7100 hydrofluoroether additive (3M Company, St. Paul, MN, USA), using an airbrush. The plants were then covered with a polyethylene frame to create a humid chamber and kept in the dark at 20–23 °C for 24 h. Afterward, the polyethylene was removed, and the plants were incubated under the same phytotron conditions. Control plants were mock-inoculated with the same carrier solution (NOVEC 7100, 3M Company, St. Paul, MN, USA)) without fungal spores and maintained in a separate climate chamber under the same controlled regime to prevent cross-contamination.

Visible stem rust symptoms were observed visually by plant pathologists from the All-Russian Institute of Plant Protection (Saint Petersburg, Russia) from the 6th to the 10th day after inoculation. The absence of visible symptoms at the earliest imaging days was confirmed by daily visual inspection prior to each hyperspectral imaging session. No symptoms were detected on any plant at the first imaging day (DAI 4 for stem rust, DAI 2 for blotch diseases). Phytopathological assessment was performed according to standard laboratory protocols for seedling processing [37]. Typically, the infection type is recorded 10–12 days after infection. On the 10th day after infection, the infection type corresponded to a score of 4 on the standard 0 to 4 modified Stakman barley scale [38] for the ‘Lyuboyar’ cultivar, indicating high susceptibility to stem rust. The severity of stem rust infection was assessed using the modified Cobb scale after Peterson [39], ranging from 70 to 100 in all conducted experiments.

Visible symptoms of spot blotch (*B. sorokiniana*) and net blotch (*P. teres*) were observed visually by plant pathologists from the All-Russian Institute of Plant Protection (Saint Petersburg, Russia) from 3 to 7 days after inoculation. Seedling infection responses were assessed on the second leaf 6–7 days after inoculation using the Tekauz 1–10 scale for *P. teres* [40] and the Fetch and Steffenson 1–9 scale for *B. sorokiniana* [41]. Infection responses were determined as 10 for *P. teres* and 9 for *B. sorokiniana* indicating high susceptibility to both pathogens. For quantitative assessment of disease development, net blotch and spot blotch severity was evaluated using the second digit of the Saari–Prescott double-digit scale [42]. Since our experiments were performed on seedlings (BBCH stage 12–13, two or three true leaves), the first digit of the scale (vertical disease progress in the canopy) was not applicable and was therefore omitted. In all conducted experiments, both diseases consistently scored 7–9, indicating severe infection.

### 2.2. Hyperspectral Imaging

Hyperspectral imaging was performed in a light-isolated room. The camera was mounted horizontally on a tripod 0.5 m above the samples. Samples were illuminated by two 500 W halogen lamps at 45°. A dark background was used as optimal for imaging plant material [43]. The frame area was 20 × 20 cm. The same labelled containers were imaged daily throughout the observation period, and each container represented an independent biological replicate.

Experiments employed the Ultris 20 hyperspectral snapshot camera (Cubert GmbH, Ulm, Germany), acquiring data in the 450–874 nm range with 106 channels at 4 nm intervals. Image resolution was 410 × 410 pixels. Calibration included black/white standards, distance calibration, and proprietary Cubert-Pilot software version 2.8.1 (Cubert GmbH, Ulm, Germany). The camera was calibrated before each imaging session according to the manufacturer’s protocol, ensuring consistent spectral performance across all experiments.

Images were taken daily at 12:00 from day 4 to day 8 post-inoculation (*P. graminis*) and from day 2 to day 6 post-inoculation (*P. teres* and *B. sorokiniana*). These intervals were chosen to span the onset of reliable classification before visible symptom development. They also reflect the different latent periods of the pathogens: *P. graminis* has a longer asymptomatic period, while symptoms of *P. teres* and *B. sorokiniana* appear earlier. The imaging intervals were also selected based on our previous binary classification experiments [17,18], which showed that reliable classification of stem rust is not achievable before DAI 4. The temporal relationship between imaging and symptom development was as follows. For *P. graminis*, imaging was performed from DAI 4 to DAI 8, while first visible symptoms appeared on DAI 6. Therefore, DAI 4 and DAI 5 were strictly pre-symptomatic for stem rust. For *P. teres* and *B. sorokiniana*, imaging was performed from DAI 2 to DAI 6, while first visible symptoms appeared on DAI 3. Therefore, DAI 2 was strictly pre-symptomatic for these diseases, and DAI 3 corresponded to the onset of symptom development.

To enable a direct comparison between pathogens with different latent periods, we introduced a relative “day” position (ranging from 1 to 5), where position 1 corresponds to the first day of imaging for each pathogen (i.e., DAI 4 for *P. graminis* and DAI 2 for *P. teres* and *B. sorokiniana*). This relative scale does not represent the absolute number of days after inoculation, but rather the ordinal position of each imaging day within the respective observation window. All reported results in Section 3.5 are expressed in this relative “day” position.

Each daily dataset contained 144 images (72 healthy, 72 diseased). Each complete dataset comprised 720 images (360 healthy, 360 diseased). Data were saved in Multi-Channel TIFF format (106 channels, 16-bit). For classification, each container-day combination was treated as one sample: the 12 images acquired per container per day were averaged into a single mean spectrum, yielding 30 samples per class per experiment (6 containers × 5 days).

### 2.3. Binary Classification Check

Before addressing the multiclass task, the datasets were validated using our previously described preprocessing pipeline [18]. This pipeline was originally designed for binary “healthy vs. diseased” early plant disease classification. Applying it to the current datasets yielded F1 values exceeding 0.89 for both datasets of all three pathogens in the binary task. This confirmed that the spectral data contained sufficient disease-related information and that the experimental material was suitable for further analysis. After this validation step, healthy samples were excluded, and the dataset was reduced to infected plants only. The multiclass classification task described below therefore operated exclusively on diseased samples.

### 2.4. Preprocessing

Before feature extraction, the curve correction and channel correction were applied sequentially. This preprocessing pipeline followed the general approach described in our previous work [18], where a similar combination of corrections proved critical for detecting asymptomatic infections. Savitzky–Golay smoothing was applied with window length and polynomial order optimized via Optuna (window length: 5, 7, 9, 11, 15, 21; polynomial order: 2, 3;). The complete pipeline, summarized in Table 1, was applied in the following order: curve-wise correction, channel-wise correction, and Savitzky–Golay smoothing.

**Table 1:** Preprocessing sequence.

Stage	Notation	Purpose
Curve correction	curve-wise/cu	Correction of each spectral vector as an individual curve to reduce the influence of the overall signal level and shape.
Channel correction	channel-wise/ch	Compensation for systematic differences between spectral channels caused by their unequal sensitivity and recording conditions.
Smoothing	Savitzky–Golay/sg	Suppression of high-frequency noise while preserving the local shape of the spectral curve before derivatives and features are calculated.

### 2.5. Feature Extraction

At the output of the preprocessing pipeline, the smoothed spectral curves were further transformed into several alternative feature representations. The purpose of this comparison was to determine which representation of the spectral signal is most informative for discriminating between the three barley diseases, rather than to rely on a single, arbitrarily chosen feature set. Four representations were evaluated: the smoothed spectrum itself, its first difference, spline-based features, and spectral indices. Table 2 summarizes these representations and provides a brief description of each representation of the spectral data that was compared at the output of the pipeline. A set of 9 vegetation indices was computed from the preprocessed spectra: NDVI, GNDVI, NDRE, PRI, SIPI, PSRI, ARI, CRI and MCARI. These indices are commonly used in plant stress and disease detection studies, as they capture specific physiological and biochemical properties of the leaf (chlorophyll content, carotenoid/anthocyanin ratios, senescence, and red-edge position). The full list of indices and their formulas is provided in Table S1 (Supplementary Materials).

**Table 2:** Feature representations.

Representation (Notation)	Description
Smoothed spectrum (savgol)	Spectral-curve values after sequential correction and smoothing.
First difference (savgol_d1)	Signal change between neighboring spectral channels; emphasizes local spectral transitions.
Spline-based features (savgol_bspline_features)	Compact description of the spectral-curve shape using a spline representation.
Spectral indices (savgol_spectral_indices)	Dimensionless combinations of channels reflecting ratios or contrasts between spectral regions.

To obtain a compact representation of the spectral data, curve approximation using a B-spline basis was employed. Each spectral curve was represented as a linear combination of basis functions:

$$\hat{x}\left(\lambda \right)=\;\Sigma_{j=1}^{q}c_{j}B_{\left(j,d\right)\left(\lambda \right)}$$

where *λ* is the spectral coordinate, *B*_(_*_j_*_,_*_d_*_)_ is the j-th basis B-spline function of degree *d*, *c_j_* is the approximation coefficient, and *q* is the number of basis functions. A common basis with uniformly spaced knots across the spectral range was used for all samples. The coefficients were determined separately for each curve using the least-squares method:

$$c*=\mathrm{argmi}n_{c}\Sigma_{i=1}^{m}{\left[x_{i}-\Sigma_{j=1}^{q}c_{j}B_{\left(j,d\right)\left(\lambda_{i}\right)}\right]}^{2}$$

where m is the number of spectral channels, and x_i_ is the value of the original curve in the channel with coordinate λ_i_. The resulting vector of coefficients was used as the feature representation of the sample:

$$z\;=\;\left(c_{1}^{*},\;c_{2}^{*},\;\ldots ,\;c_{q}^{*}\right)$$

Thus, the original spectrum consisting of *m* values was replaced by a set of *q* features characterizing its shape. Class labels were not used during basis construction or coefficient calculation. The flexibility of the approximation was determined by the number of knots and the spline degree. The number of knots was selected within the range of 5 to 12, while the spline degree was chosen from the values 2 and 3. The spline parameters for the configuration are included in the Optuna search space. The basis was constructed using the `SplineTransformer` from the scikit-learn library, retaining all basis functions.

The notation used for each representation corresponds to the configuration names reported in the experimental results. Mean spectral curves were constructed separately for each class and experimental combination to visualize the effect of preprocessing and to assess the magnitude of inter-experimental variability. To avoid information leakage, all parameters estimated from the data, including those used for spline fitting, index calculation, and curve normalization, were fitted exclusively on the training portion of the corresponding split.

### 2.6. Machine Learning Models

The preprocessing and classification pipeline previously described in [18] was initially applied to the current dataset. However, this pipeline was designed for binary “healthy vs. diseased” classification. It failed to achieve satisfactory performance in the multiclass setting, with F1 scores dropping to 0.55–0.60.

We therefore re-evaluated the feature extraction step and introduced new features, which provided a more compact and flexible description of the spectral curve shape (Section 2.5). This change led to a marked improvement in classification performance. Among the previously used models, Logistic Regression proved the most effective, while Random Forest, LightGBM and SVM still showed inferior results. We therefore retained Logistic Regression as the best of the original classifiers and additionally evaluated two alternative models, PLS-DA and kNN, which had demonstrated strong performance in similar hyperspectral classification tasks in plant pathology [44,45,46].

Logistic regression (LogReg) is a linear probabilistic model that estimates an object’s class membership using a logistic function; in the multiclass case, a set of linear scores is used for all classes. The model is compact and highly interpretable, but assumes that the separating relationship in the selected feature space is approximately linear [47].

Partial Least Squares Discriminant Analysis (PLS-DA) is a discriminant-analysis method based on partial least squares. It constructs a small number of latent components while accounting simultaneously for variation in the spectral features and their relationship to the class encoding; this is particularly useful for correlated, high-dimensional spectral features [48].

K-nearest neighbors (kNN) is a nonparametric algorithm that assigns an object to the class represented by the majority of its nearest neighbors in feature space. It does not impose a global boundary shape and is therefore sensitive to scale, point density, and inter-experiment shift [49].

Hyperparameters for each classifier were optimized using the Optuna library with a TPE sampler [50]. The search space for each model is summarized in Table 3. The objective function was the mean validation accuracy across five validation splits.

**Table 3:** Hyperparameters of the investigated models and their optimization spaces.

Model	Hyperparameter	Description	Search Range
Savitzky–Golay Filter	window_length	Smoothing window length	5, 7, 9, 11, 15, 21; categorical variable
	polyorder	Polynomial degree	2, 3; categorical feature
Spline	n_knots	Number of knots	integers from 5 to 12
	degree	Spline degree	2, 3
Logistic regression	C	A parameter inversely proportional to the regularization strength;	From 10^−5^ to 5, logarithmic scale
	penalty	Regularization type: L2 penalty	L2, None
	max_iter	Maximum number of optimization algorithm iterations during training	Integers from 50 to 500
PLS-DA	n_components	Number of latent components	Integers from 1 to 10
	max_iter	Maximum number of algorithm iterations	Integers from 100 to 1000
kNN	n_neighbors	Number of nearest neighbors	Integers from 1 to 10
	weights	Neighbor weighting method: equal weights (uniform) or weights inversely proportional to distance (distance)	uniform, distance
	p	Minkowski metric parameter: Manhattan distance is used when p = 1, and Euclidean distance when p = 2.	1; 1.5; 2; 2.5; 3

### 2.7. Training, Validation, and Testing Scheme

The dataset consisted of six independently imaged experiments: two for each disease—net blotch (NB1, NB2), spot blotch (SB1, SB2), and stem rust (SR1, SR2). The splitting was performed at the experiment level, not at the level of individual images. Therefore, images from the same experiment never appeared simultaneously in both training and testing sets.

As shown in Table 4 and Table 5, for each disease, one experiment was fully held out for testing, while the other was used for training. This corresponds to a leave-one-out procedure by experiment, applied independently to each disease. All 2 × 2 × 2 = 8 possible combinations were enumerated. Formally, for *i*, *j*, *k* ∈ {1, 2}

*D* = *NB*_1_ ∪ *NB*_2_ ∪ *SB*_1_ ∪ *SB*_2_ ∪ *SR*_1_ ∪ *SR*_2_

**Table 4:** Day-wise cross-validation.

Split	Training	Cross-Validation
D_1_	D_2_, D_3_, D_4_, D_5_	D_1_
D_2_	D_1_, D_3_, D_4_, D_5_	D_2_
D_3_	D_1_, D_2_, D_4_, D_5_	D_3_
D_4_	D_1_, D_2_, D_3_, D_5_	D_4_
D_5_	D_1_, D_2_, D_3_, D_4_	D_5_

**Table 5:** Experiment-wise testing.

Split	Training	Test
E_1_	NB_1_, SB_1_, SR_1_	NB_2_, SB_2_, SR_2_
E_2_	NB_1_, SB_1_, SR_2_	NB_2_, SB_2_, SR_1_
E_3_	NB_1_, SB_2_, SR_1_	NB_2_, SB_1_, SR_2_
E_4_	NB_1_, SB_2_, SR_2_	NB_2_, SB_1_, SR_1_
E_5_	NB_2_, SB_1_, SR_1_	NB_1_, SB_2_, SR_2_
E_6_	NB_2_, SB_1_, SR_2_	NB_1_, SB_2_, SR_1_
E_7_	NB_2_, SB_2_, SR_1_	NB_1_, SB_1_, SR_2_
E_8_	NB_2_, SB_2_, SR_2_	NB_1_, SB_1_, SR_1_

The test and training sets were then defined as:

$$D_{\mathrm{test}}^{i,j,k}=\;NB_{i}\cup \;{\mathrm{SB}}_{j}\cup \;SR_{k}$$

$$D_{\mathrm{train}}^{i,j,k}=D-D_{\mathrm{test}}^{i,j,k}$$

The eight test splits corresponded to all possible combinations of indices i, j, and k, as summarized in Table 4 and Table 5. The eight splits are not independent replicates: they overlap in the experiments they contain, and each experiment appears in the test set in four of the eight splits. The mean, standard deviation, and range across the eight splits should therefore be interpreted as a measure of variability across experimental combinations, not as a formal statistical estimate. The resulting splits were saved in a tabular-dataset format compatible with the comparative testing framework [51].

Table 4 shows the day-wise cross-validation scheme used for hyperparameter tuning within each training portion. The right column shows the held-out cross-validation day (D1–D5); the remaining four days were used for training. The held-out day changed successively from D1 to D5.

Table 5 shows the experiment-wise test splits used for final evaluation. The right column shows experiments held out for testing. Split numbers 1–8 correspond to all possible combinations of NB, SB, and SR experiments in testing (2 × 2 × 2 = 8). NB, net blotch; SB, spot blotch; SR, stem rust.

Within each training portion, hyperparameters were tuned using five validation splits by imaging day. At each step, one day was used for validation while the remaining days were used for training only. The held-out day changed successively from D_1_ to D_5_; for testing, the fully held-out experiment similarly changed from E_1_ to E_8_. Thus, validation never used any images from the three experiments held out for the corresponding test. It should be noted that samples from the same container are not statistically independent, since each container was imaged on all five days. The day-wise cross-validation scheme therefore evaluates generalization to new imaging days rather than to new containers. This is the intended design: in a controlled experiment, all containers are prepared identically, and container identity is not a meaningful source of biological variation. Container-level leakage between training and testing was avoided by splitting at the experiment level. Preprocessing and all data-dependent transformations were refitted exclusively on the current training portion. After the configuration was selected, it was fixed and evaluated separately on each of the eight test splits. The mean, standard deviation, and range of the balanced accuracy across the eight experiment-level test splits were then reported.

Because the dataset was balanced and contained an equal number of objects in each class, ordinary accuracy and balanced accuracy are numerically identical in this study. We therefore use them interchangeably, and report balanced accuracy throughout the manuscript to make the class balance explicit. Ordinary accuracy is defined as:

$$\mathrm{Accuracy}\;=\frac{1}{N}\sum_{i=1}^{N}I\left(\hat{y_{i}}=\;y_{i}\right)$$

where *N* is the number of objects, *y_i_* is the true class, and $\hat{y_{i}\;}$ is the predicted class. Since the classes were equally represented, ordinary accuracy was not distorted by differences in class size.

## 3. Results

This section presents the results of the multiclass classification of three barley diseases using hyperspectral imaging and machine learning. Firstly, the structure of the dataset was described and the balance of the test splits was verified. The performance of different classifiers and feature representations was then compared, the best configuration was identified, and its performance was analyzed across imaging days and disease classes. Finally, the resulting confusion matrix was examined.

### 3.1. Sample Distribution

**Figure 1:**
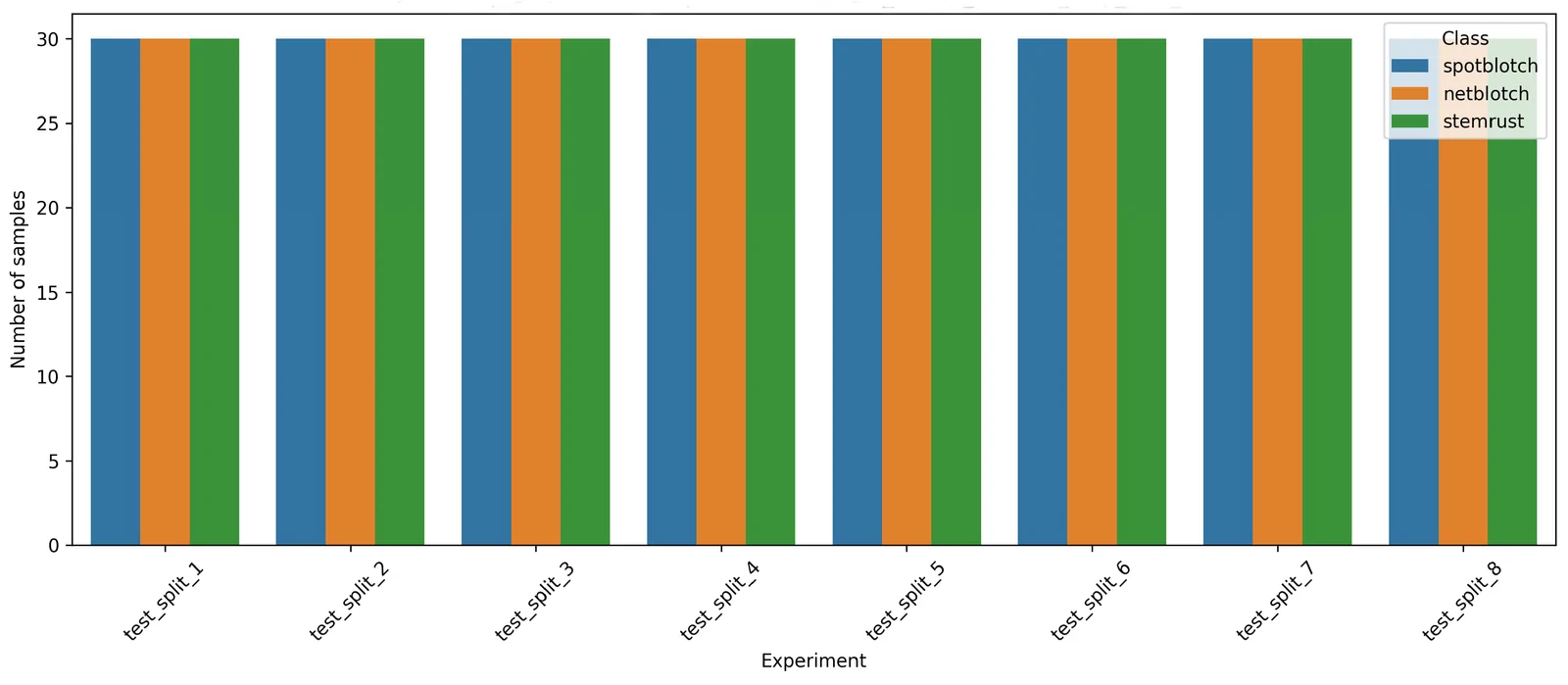
Distribution of hyperspectral image samples across the eight test splits and three disease classes (spot blotch, net blotch, stem rust). Each test split contains an equal number of samples per class, confirming that the test groups are balanced with respect to the target variable and that differences in accuracy reflect the difficulty of disease recognition rather than class size.

Figure 1 shows the structure of the dataset. Each experiment included five imaging days, six unique plant boxes, and 72 hyperspectral images per day (12 images per box, later averaged into one sample per box-day), yielding a total of 2160 images across all experiments. Only images of infected plants were used for the multiclass classification task; healthy samples were employed solely for the preliminary validation step described in Section 2.3. In each of the eight test splits, the three classes—spot blotch, net blotch, and stem rust—were represented by an equal number of images. Therefore, the test groups were balanced with respect to the target variable, and differences in accuracy between models and configurations reflect the difficulty of recognizing the diseases rather than class size. This balance ensures a fair comparison across all tested combinations. In each of the eight test splits, the three classes were represented by an equal number of samples (30 per class), each corresponding to one container-day combination.

### 3.2. Spectral Curves After Preprocessing

The mean spectral curves of the three classes after each preprocessing stage are shown in Figure S2a–f (Supplementary Materials S3). In the raw spectra (Figure S2a,b), the overall level and shape differed substantially between experiments, reflecting the influence of external factors such as illumination, plant age, and leaf position; the differences between the three classes were relatively small compared to the inter-experimental variability.

After curve correction (Figure S2c,d), the curves became more comparable in overall level, and class-specific differences became more visible, particularly in the red-edge region (~700–750 nm). However, inter-experimental variability persisted. After curve and channel correction (Figure S2e,f), the systematic differences between spectral channels were compensated, and the curves became more sensitive to local spectral features, particularly in the green peak (~550 nm) and red-edge regions. At the same time, cu + ch correction amplified high-frequency variation and inter-experimental variability, especially in the 500–600 nm and 700–800 nm regions.

This observation has two important implications. First, it confirms that random splitting of the data would lead to over-optimistic results, because the model could learn to associate experimental artefacts with disease classes. Second, it explains why more complex classifiers (such as kNN) underperformed: their distance metrics are highly sensitive to the amplified high-frequency variation. In contrast, linear models with built-in regularisation (PLS-DA, LogReg) and spline-based features, which smooth the curve shape, were better able to extract stable, class-specific information from the noisy preprocessed spectra.

### 3.3. Model Selection Effect

**Figure 2:**
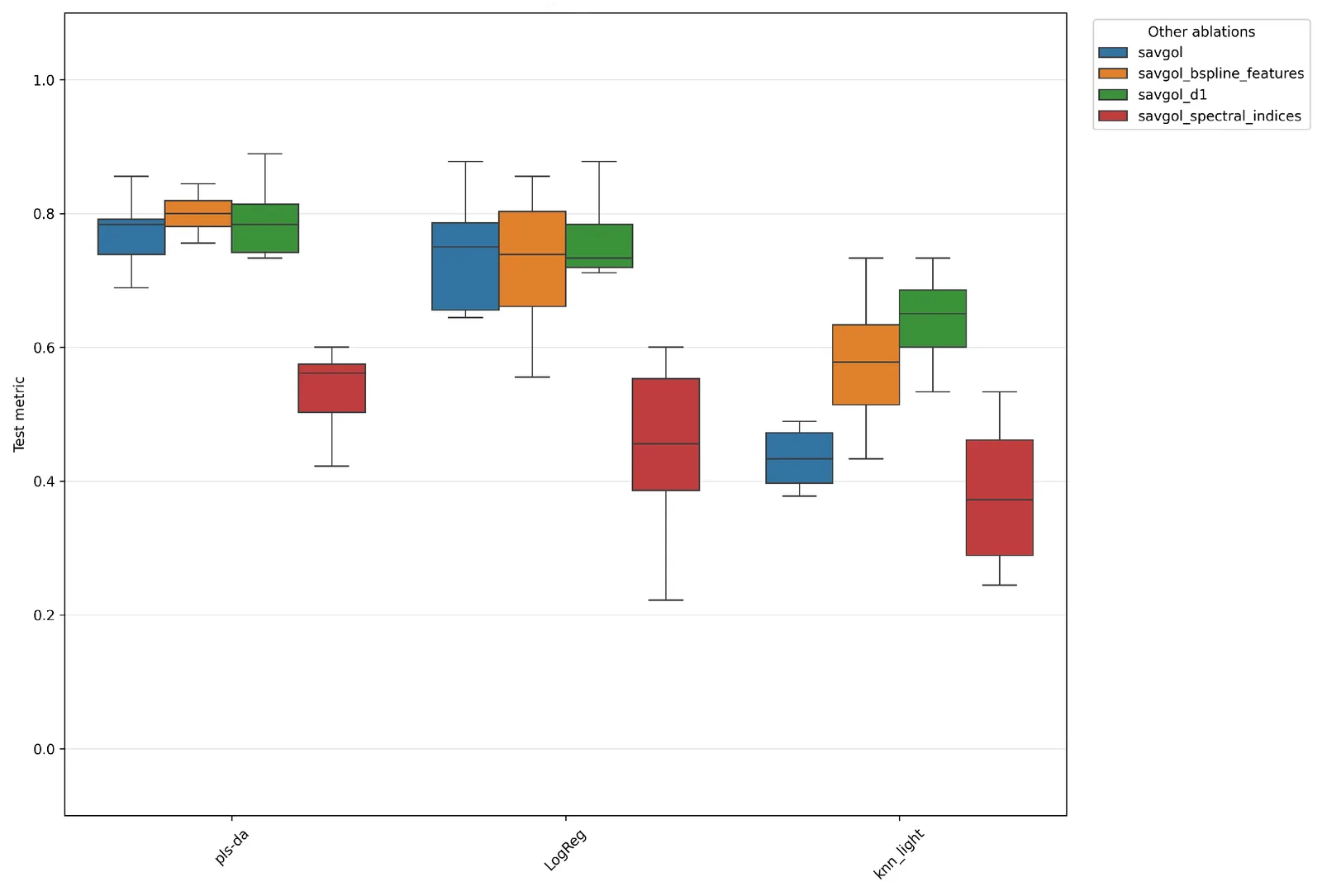
Comparison of classifier performance across different feature representations. Balanced accuracy for each classifier (PLS-DA, LogReg, kNN) is shown for the four feature representations: smoothed spectrum (savgol), spline-based features (savgol_bspline_features), first difference (savgol_d1), and spectral indices (savgol_spectral_indices). Each box represents the distribution across eight test splits. The boxes show the median and interquartile range; whiskers extend to the full range of the data. The y-axis shows balanced accuracy. PLS-DA and LogReg consistently outperform kNN, and spectral indices consistently underperform compared to the other representations.

**Figure 3:**
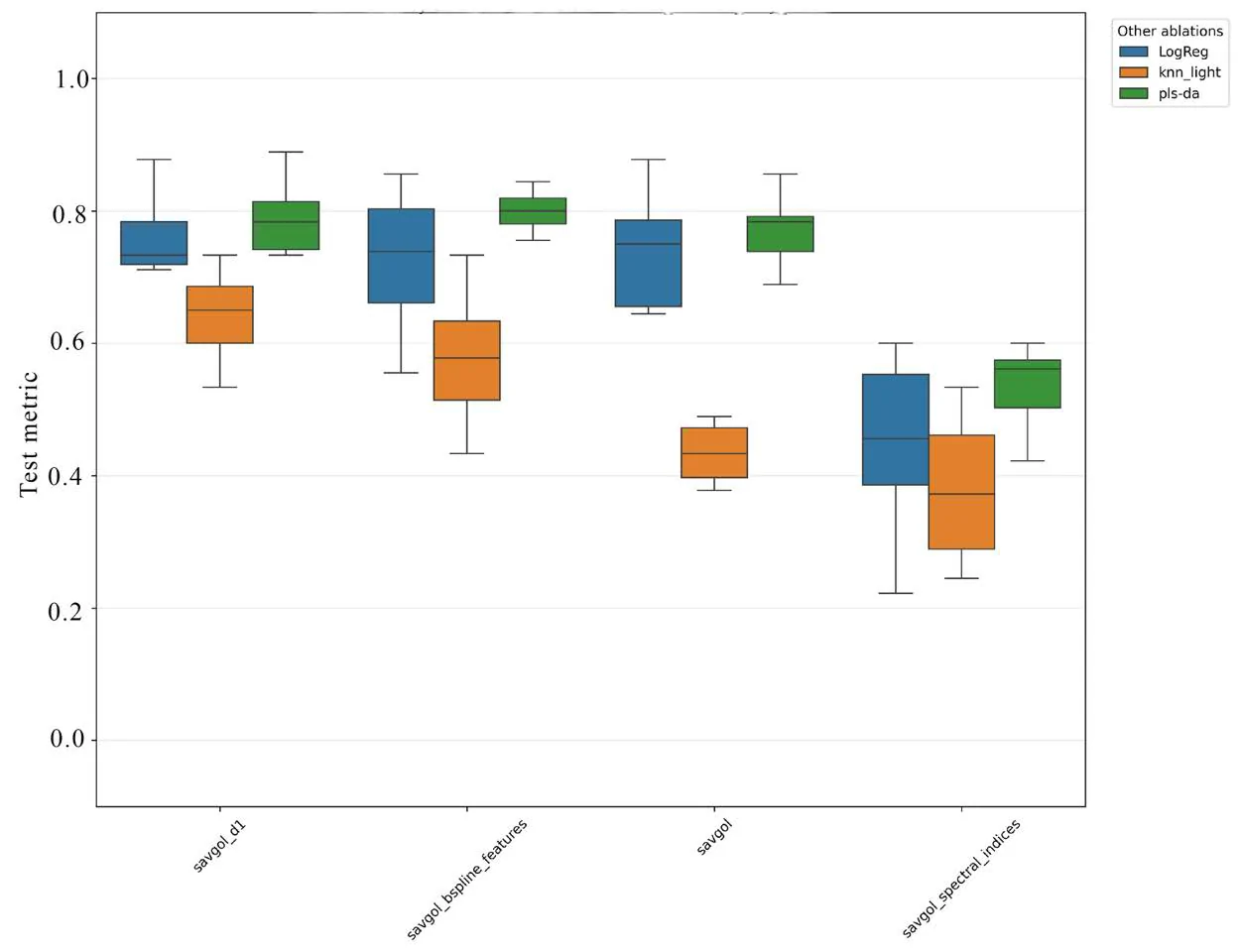
The same results as in Figure 2, grouped by feature representation instead of classifier. Balanced accuracy is shown for each feature representation. The boxes show the median and interquartile range; whiskers extend to the full range of the data. The y-axis shows balanced accuracy. PLS-DA and LogReg consistently outperform kNN, and spectral indices consistently underperform compared to the other representations.

Figure 2 and Figure 3 compare the performance of the three classifiers across all feature representations. When averaged across all four feature representations, the mean accuracy across the presented combinations was 0.725 for PLS-DA, 0.667 for Logistic Regression, and 0.512 for k-NN.

The kNN classifier underperformed the linear models in nearly all feature representations, with the lowest values observed for the smoothed spectrum and spectral indices. A possible explanation is that hyperspectral features are high-dimensional and exhibit a pronounced inter-experiment shift: as the distance between samples is increasingly dominated by experimental variation rather than biological differences, the nearest-neighbour metric becomes less informative. In addition, kNN is sensitive to feature scaling and point density, which further reduces its robustness in this setting. The larger spread of kNN results across test splits (taller boxes in Figure 3) supports this interpretation.

In contrast, PLS-DA and Logistic Regression were able to extract a more stable global boundary between classes. PLS-DA showed slightly higher mean accuracy across most feature representations, which may be explained by its ability to construct latent components that simultaneously account for spectral variation and class separation. This is particularly useful when the number of features is large and the features are highly correlated—a typical situation for hyperspectral data.

It is also worth noting that the choice between index-based and curve-based feature representations had a stronger effect on performance than the choice between linear classifiers. For example, the difference between PLS-DA with spline features and PLS-DA with spectral indices was larger than the difference between PLS-DA and Logistic Regression for the same representation. However, among the non-index representations, the differences between classifiers and feature types were smaller and partly within the observed variability. This suggests that avoiding information-poor representations (such as spectral indices) is more important than fine-tuning the choice of classifier or non-index feature type. These trends are examined next at the level of individual model–feature combinations, using the full ablation across all eight test splits.

### 3.4. Best Model

**Figure 4:**
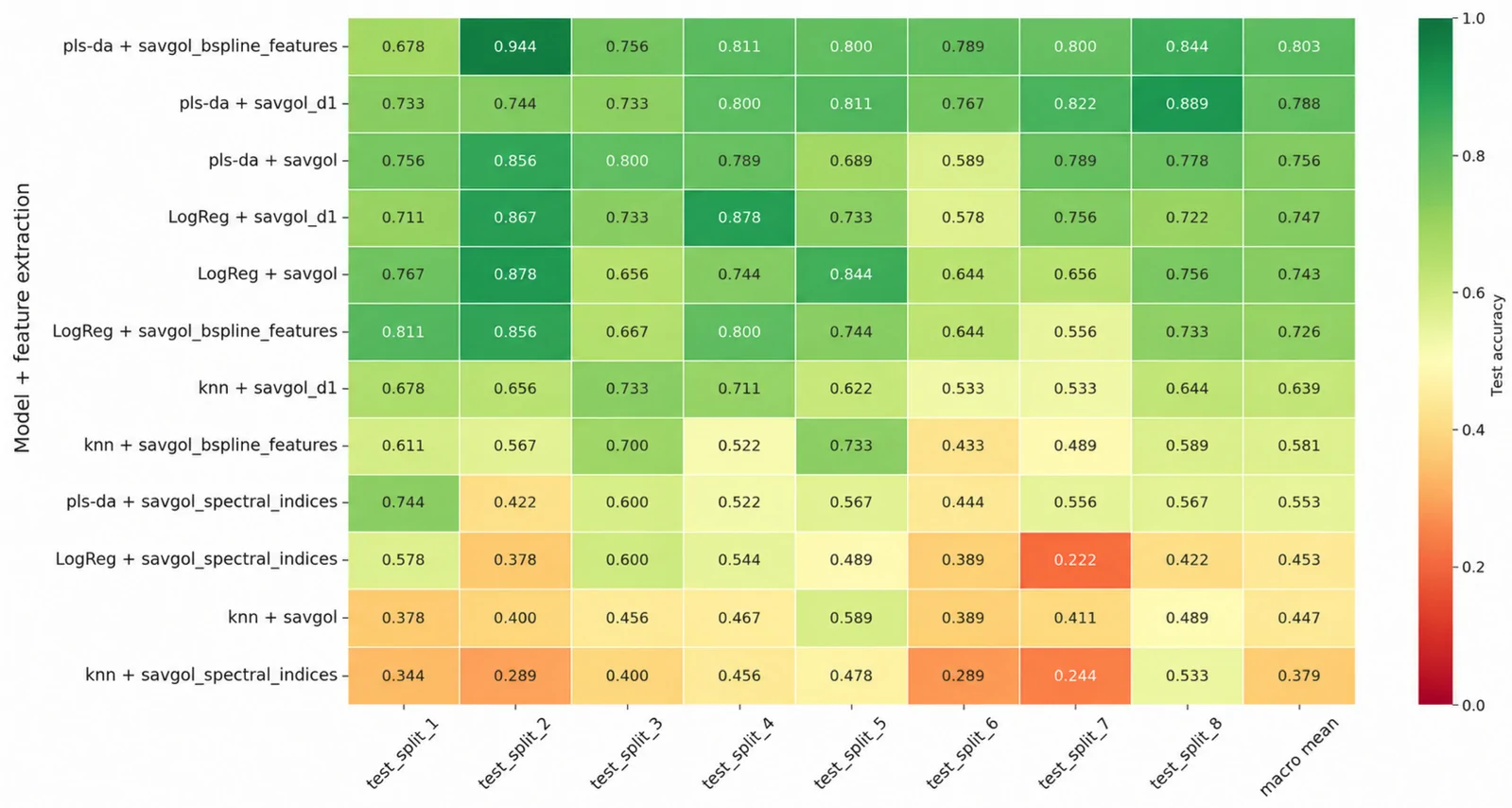
Mean balanced accuracy for all combinations of model and feature representation across the eight experiment-level test splits. Each row corresponds to one configuration (model + features), each column to one test split; the last column shows the mean across the eight splits. The color scale reflects balanced accuracy from 0.0 (red) to 1.0 (dark green). The best-performing configuration was PLS-DA with spline-based features (mean balanced accuracy = 0.803).

The complete ablation across the eight test splits (Figure 4) identified the configuration PLS-DA with spline-based features as the best-performing combination, with a mean balanced accuracy of 0.803 across the eight splits. The second-best result was obtained by PLS-DA with first-difference features (0.788). Both configurations are based on a compact representation of the spectral curve shape, which suggests that the form of the curve—rather than the absolute reflectance values—is the most informative feature for distinguishing between the three pathogens.

The spread of accuracy across the eight test splits was considerable: for the best configuration, it ranged from 0.678 (split 1) to 0.944 (split 2), with a standard deviation of 0.076. This indicates that some experimental combinations were substantially easier to classify than others. Importantly, even the lowest-performing split (0.678) remained well above the random-chance level (0.333), confirming that the model captures genuine biological signal rather than experimental artefacts.

Configurations based on spectral indices consistently underperformed. This suggests that spectral indices, being dimensionless combinations of channels, lose the shape information that is critical for distinguishing between pathogens with similar physiological effects.

Configurations based on k-NN consistently underperformed the linear models. This is consistent with the conclusion in Section 3.3: k-NN is sensitive to inter-experimental shift, because its distance metric depends on the global structure of the feature space.

Finally, transforming the spectral curve via differentiation or splines yielded a consistent improvement over the raw smoothed spectrum. This supports the hypothesis that, for early disease differentiation, local features of the spectral curve shape are more important than absolute reflectance values. The analysis so far has been aggregated across imaging days. We next examine how the performance of the best configuration changes over the observation period.

### 3.5. Accuracy Distribution by Day

**Figure 5:**
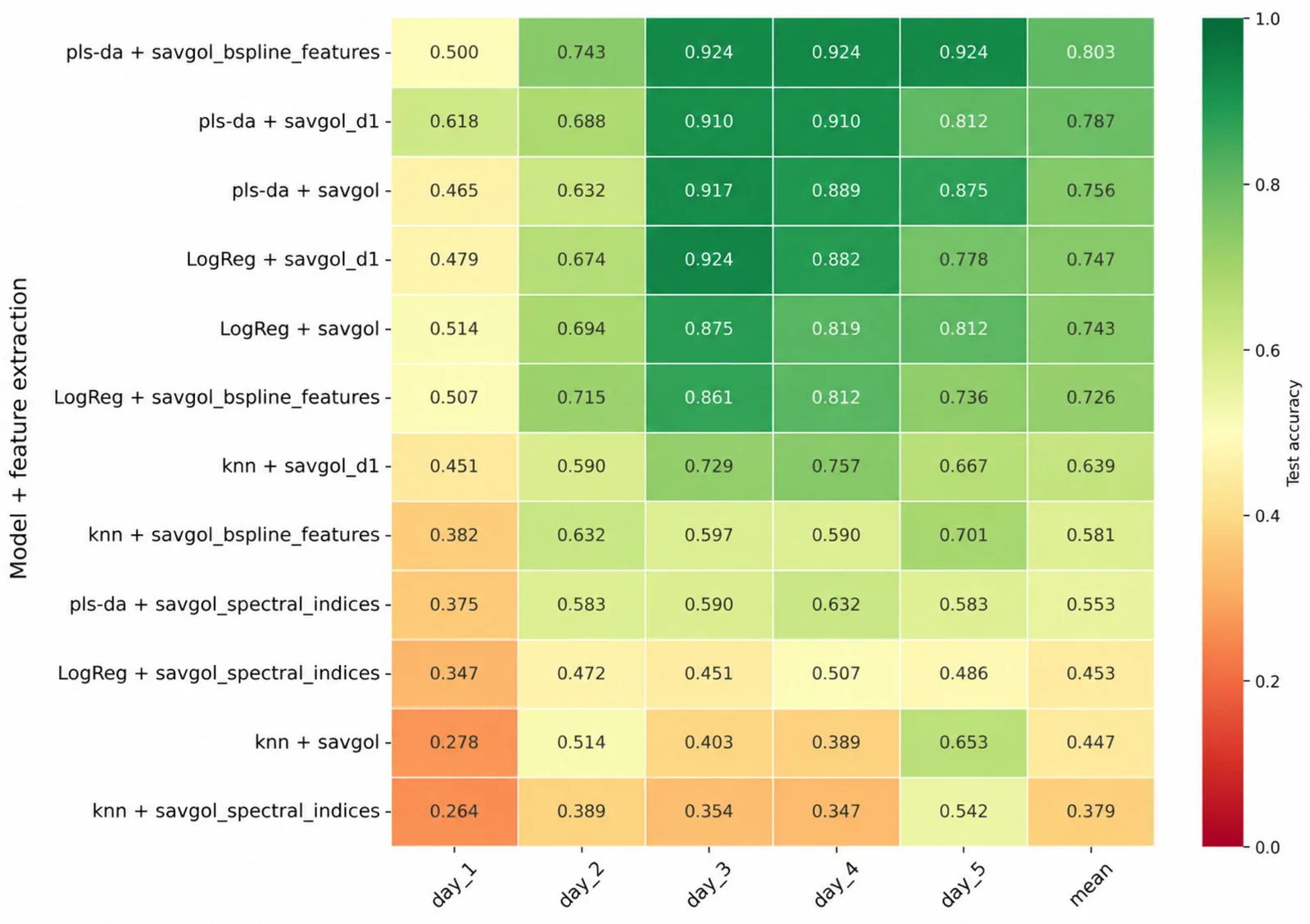
Mean balanced accuracy by relative imaging day for all model–feature combinations. Each row corresponds to one configuration, each column to one imaging day (Day 1–Day 5); the last column (mean) shows the average across days. The color scale reflects balanced accuracy from 0.0 (red) to 1.0 (dark green). The best configuration (PLS-DA with spline-based features) shows a sharp drop on Day 1 (0.500), followed by a rapid increase and a plateau of 0.924 from Day 3 onward.

Figure 5 shows the accuracy of the best configuration as a function of imaging days. In this section, “Day” position refers to the relative imaging day within the observation window (1 = first imaging day), as defined in Section 2. A sharp drop in performance was observed on the first imaging day, with a mean accuracy of 0.500—only slightly above the random-chance level of 0.333 for three classes, and thus insufficient for reliable classification. Accuracy increased to 0.743 on the second day and reached a plateau of 0.924 from the third day onward, remaining stable through the fifth day. This pattern was consistent across most configurations, although the absolute values differed across configurations. In contrast, configurations based on spectral indices and kNN remained below 0.600 even on Days 3–5.

It is important to emphasize that the overall balanced accuracy of 0.803 is a mean across eight test splits and five imaging days. It conceals substantial variation: for the best configuration, accuracy ranged from 0.678 to 0.944 across splits, and from 0.500 on Day 1 to >0.900 from Day 3 onward. Therefore, the overall value should not be interpreted as a uniform performance level.

The fact that the model performs slightly better than the chance level on Day 1 is not necessarily a limitation: it reflects the biological reality that, at this stage, the pathogens have not yet induced detectable spectral changes. The rapid improvement from Day 2 onward suggests that the asymptomatic window during which classification is unreliable is relatively narrow under controlled conditions, which is an encouraging result for practical applications.

**Figure 6:**
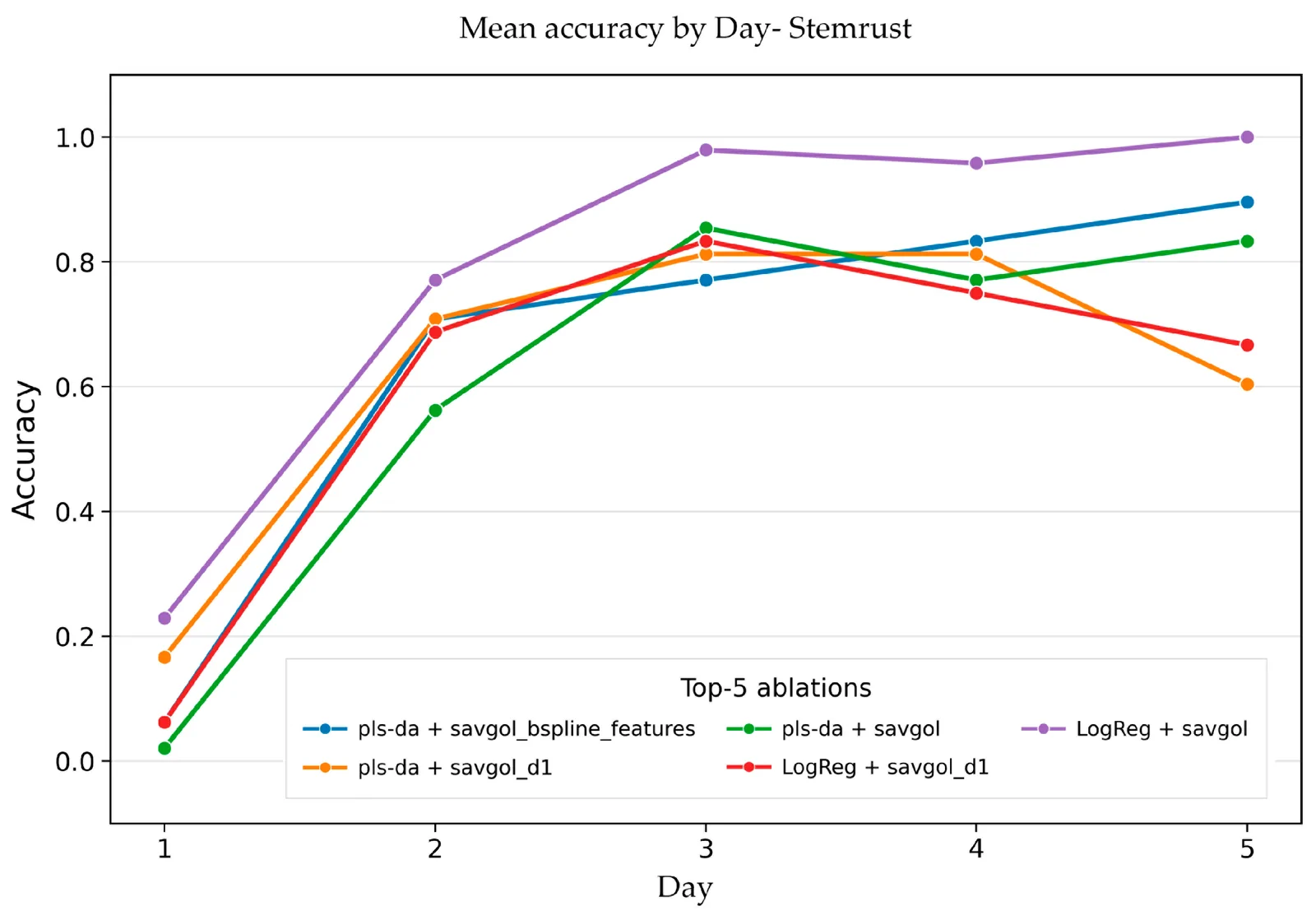
Mean class-balanced accuracy by relative imaging day for the five most successful configurations, shown for stem rust. Accuracy is close to zero on Day 1 and increases rapidly thereafter, reaching a plateau from Day 3 onward. Configurations are indicated in the legend.

**Figure 7:**
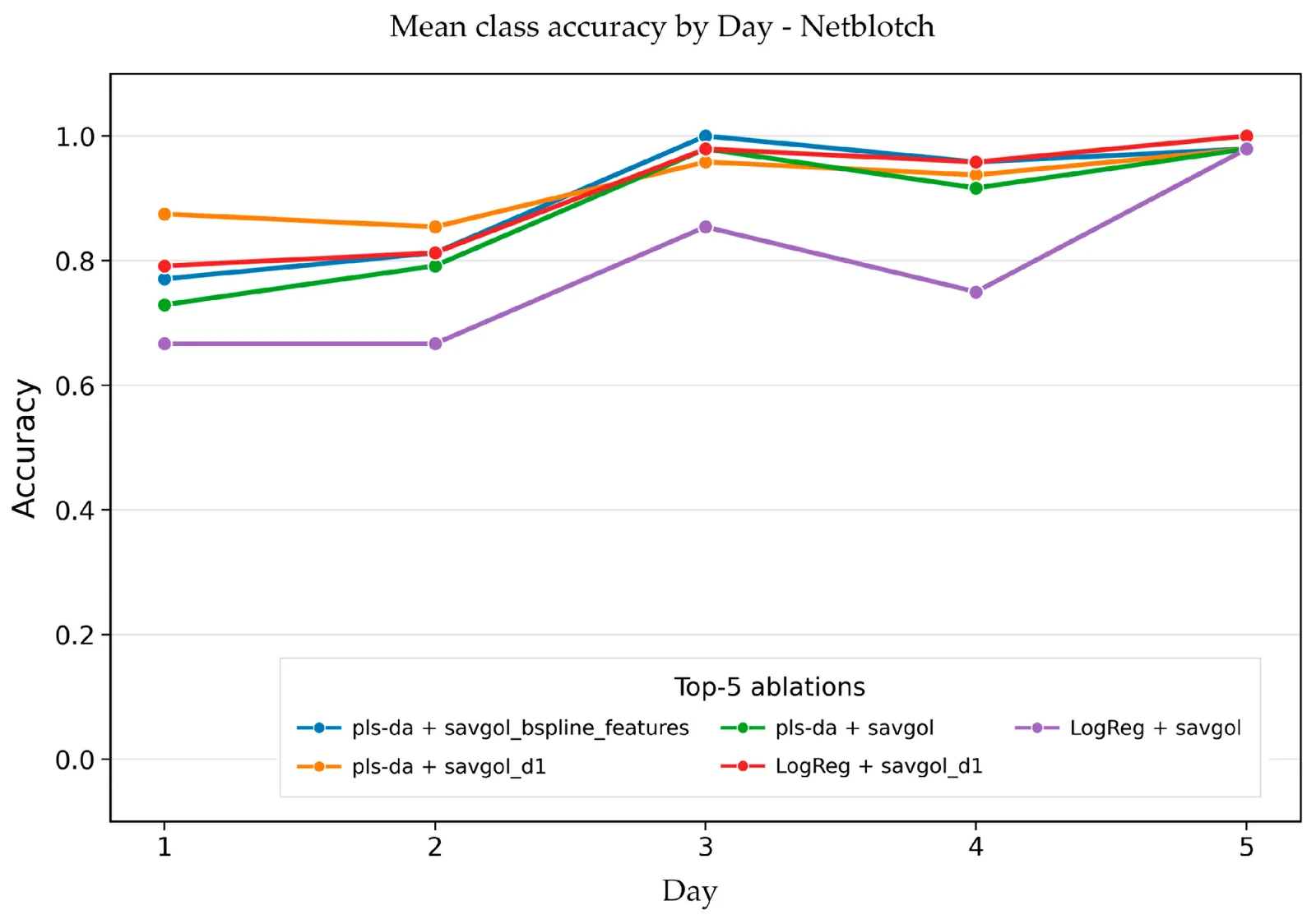
Mean class-balanced accuracy by relative imaging day for the five most successful configurations, shown for net blotch. Performance is high and stable across all five days, with a slight peak on Day 3.

**Figure 8:**
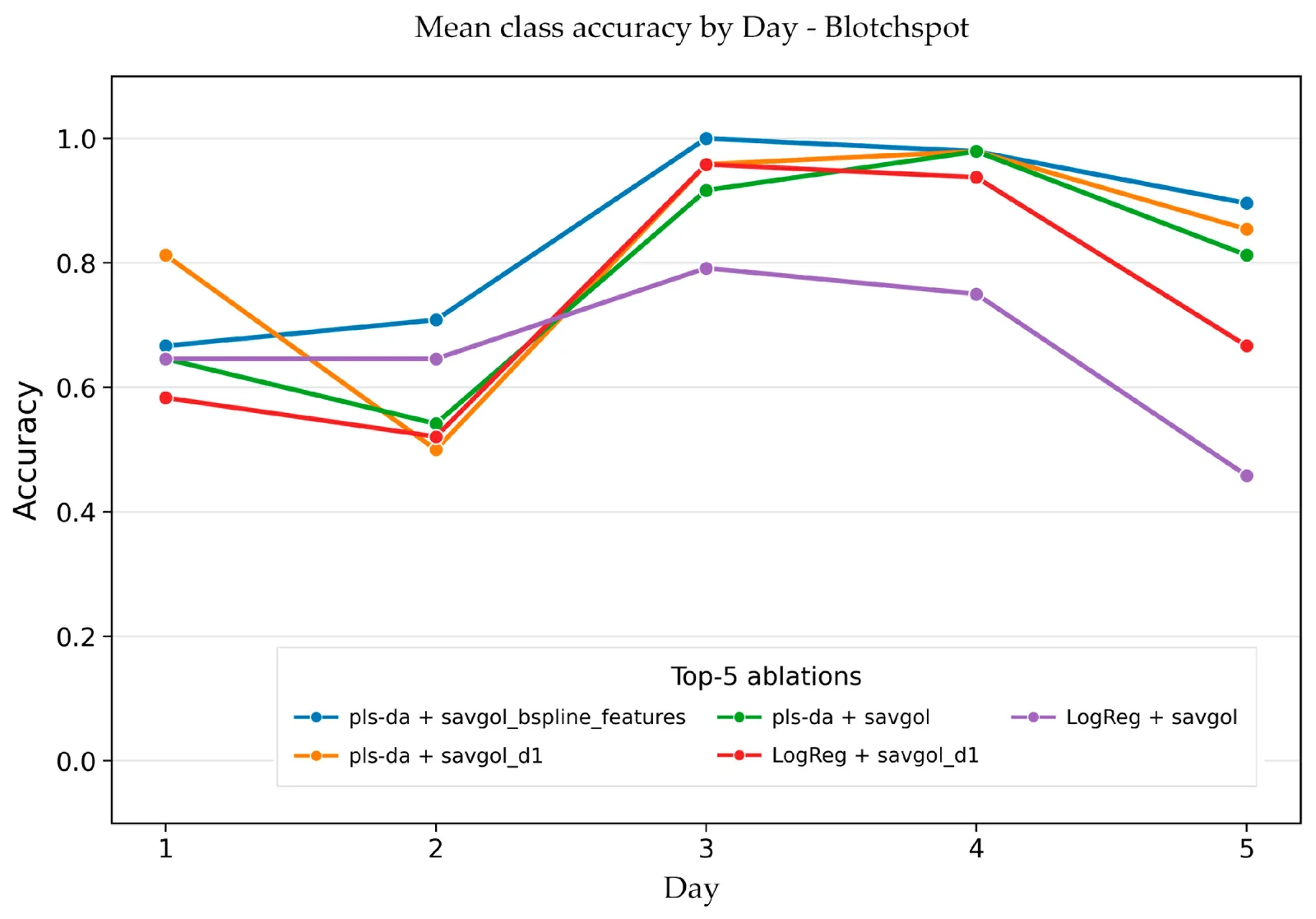
Mean class-balanced accuracy by relative imaging day for the five most successful configurations, shown for spot blotch. A dip is observed on Day 2, followed by recovery to above 0.9 on Days 3–4.

Figure 6, Figure 7 and Figure 8 provide further detail by class for the five most successful configurations. The strongest dependence on relative day was observed for stem rust (Figure 6): on the first day, the accuracy of most models was close to zero, after which it increased rapidly, reaching a plateau above 0.8 from Day 3 onward. Net blotch (Figure 7) showed a more stable pattern, with high accuracy already on Day 1 and a slight peak on Day 3. Spot blotch (Figure 8) exhibited a dip on Day 2 (down to ~0.5 for most configurations) before recovering to above 0.9 on Days 3–4.

These differences confirm that averaging across classes and days can conceal different rates at which the spectral features of different diseases develop. In particular, stem rust requires more time to develop a stable spectral signature, whereas net blotch and spot blotch become distinguishable earlier. The reason for this difference remains unclear. To examine these class-specific differences in more detail, we next turn to the confusion matrix for the best configuration.

### 3.6. Confusion Matrix

**Figure 9:**
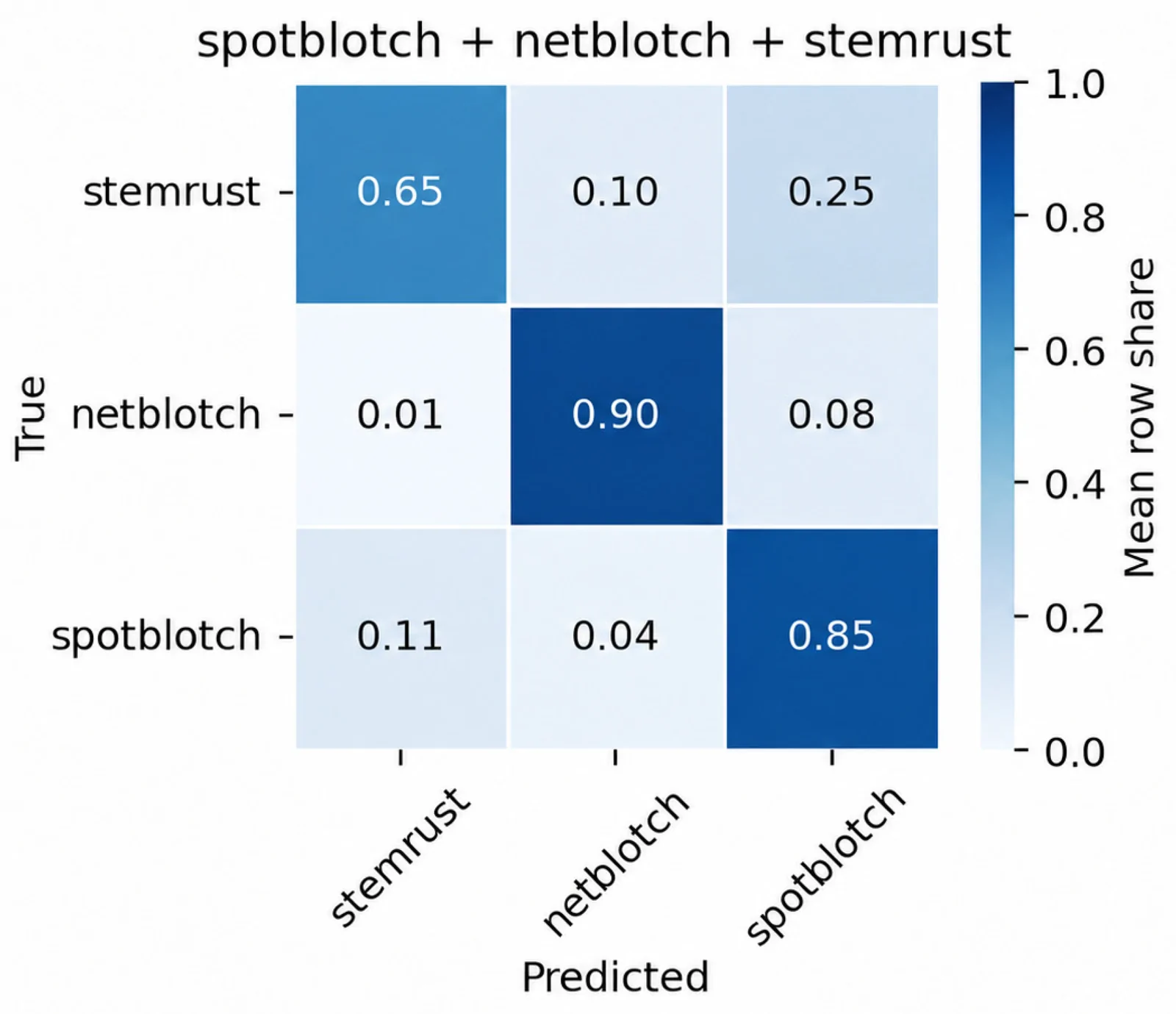
Mean normalized confusion matrix of the best configuration (PLS-DA with spline-based features) across the eight test splits. Values are normalized by the true class (rows), so each row sums to 1.0. The diagonal represents correct classifications; off-diagonal values show misclassifications. Stem rust has the lowest recall (0.65), with 25% of its samples misclassified as spot blotch.

Figure 9 shows the normalized mean confusion matrix for the best configuration (pls-da + savgol_bspline_features) across the eight test splits. Class recall was 0.850 for spot blotch, 0.904 for net blotch, and 0.654 for stem rust. Consequently, stem rust is the main source of reduced overall accuracy.

The confusion matrix reveals a pronounced asymmetry in misclassification patterns. While 24.6% of stem rust samples were misclassified as spot blotch, only 10.8% of spot blotch samples were misclassified as stem rust. Net blotch was recognized most accurately, with only 1% of its samples misclassified as stem rust and 8% as spot blotch. This suggests that stem rust is the most difficult class to distinguish, particularly in experiments where its spectral signature overlaps with that of spot blotch.

The lower recall for stem rust (0.654) compared to net blotch (0.904) and spot blotch (0.850) is the main factor reducing the overall accuracy. The reason for the lower recall of stem rust remains unclear. Possible explanations include differences in the duration of the asymptomatic period or in the biochemical composition of the pathogens, but neither can be confirmed from the present data.

The weakest test splits corresponded to cases where the recall of both stem rust and spot blotch decreased simultaneously, indicating a transferable shift between experiments rather than a single-class error. This highlights the importance of cross-experimental validation: a model that performs well on average may still fail in specific experimental conditions, and the confusion matrix provides a more detailed picture of where the errors occur.

Overall, the confusion matrix confirms that stem rust remains the main challenge for multiclass differentiation, and improving its transferability between experiments is the primary direction for further method development.

## 4. Discussion

The binary classifier “diseased vs. healthy”, based on our previous work, achieved an average F1 score above 0.89 for all three pathogens. In the multi-class task, the average balanced accuracy was notably lower (0.803). This drop is not unexpected and likely reflects the biological nature of early-stage infection. At the initial stages of pathogenesis, visible chlorosis or necrosis has not yet developed, and the host plant’s response—as well as the manifestations of early chlorosis—is remarkably similar across all three pathogens [52,53,54]. Spectral differences between pathogens at this stage are minimal, making the task of differentiating them fundamentally more complex than detecting the presence of infection. The binary task addresses the question of whether the disease is present, whereas the multi-class task asks which specific pathogen is involved. This requires a much more subtle spectral signal. Thus, the decrease in accuracy from 0.900 to 0.800 should be viewed not as a methodological shortcoming, but as a natural reflection of the task’s inherent complexity. Moreover, maintaining an accuracy above 0.800 under these conditions demonstrates that distinct differences between pathogens do exist and can be detected by machine learning models.

On the first imaging day, the average accuracy of the best configuration was 0.500, only slightly above random guessing for three classes (0.333). This indicates that spectral differences between pathogens had not yet developed sufficiently for reliable classification. The first imaging day was strictly pre-symptomatic for all three pathogens: visible symptoms of spot blotch and net blotch appear 3–4 days after inoculation, while stem rust symptoms appear after 5–6 days [55,56,57]. Therefore, the accuracy achieved on this day (0.500 on average, and higher for net blotch and spot blotch) represents classification performance in the complete absence of visible symptoms. A sharp increase in accuracy is observed by the second day (0.743), and from the third day onwards, it plateaus above 0.900. A plausible interpretation is that the pathogen requires time to accumulate sufficient biomass and to induce detectable changes in the leaf’s pigment complex [55,56,57]; however, this remains an interpretation rather than a demonstrated mechanism. At the same time, the laboratory conditions should be viewed as the most challenging scenario for early differentiation. In the field, the asymptomatic period for all three infections is typically much longer due to suboptimal temperature, humidity, and light conditions [52,53,54,55,56,57]. While the first two or three days of imaging in the laboratory fall within the early phase, this period can extend to four, six, or more days in the field. This implies that, under field conditions, the model would operate in a more favorable context than in our experiments. Notably, the low accuracy for stem rust on the first imaging day is also consistent with our previous binary classification results, where the F1-score on the first imaging day was only 0.62–0.83, compared to 0.89–0.94 on subsequent days [18]. In the multiclass case, however, the model does not simply fail to distinguish stem rust—it systematically misclassifies it, most often as spot blotch (Figure 9). This does not imply that the two pathogens are spectrally similar in principle, since their infection strategies are fundamentally different (Section 1). Rather, it suggests that at the earliest stage, the spectral signature of stem rust is too weak to be reliably distinguished, and the model defaults to the class with the more developed spectral response.

A key feature of our evaluation framework was the use of eight test splits based on experiments rather than individual images. With random splitting, images from the same experiment could end up in both the training and test sets, leading to group-level data leakage. Recent methodological studies indicate that such group leakage results in systematically inflated performance metrics [58,59,60]. Earlier methodological studies treated data splitting primarily as a matter of proportion and focused on optimizing this ratio, determining how many samples to allocate to the training set versus the test set [61,62]. However, such an approach assumes that samples within each subset are independent and fails to account for the group structure characteristic of biological data. These recommendations are inapplicable to our dataset, where each experiment represents a unique combination of conditions: no specific split ratio can prevent data leakage if images from the same group end up in both subsets. Our scheme, in which an entire experiment is consistently excluded from the training set during testing, directly addresses the issue of group-level data leakage and establishes a more appropriate evaluation standard for data with a pronounced group structure. It should be noted, however, that the eight test splits are not independent replicates: they overlap in the experiments they contain. The mean, standard deviation, and range across the eight splits are therefore reported as a measure of variability across experimental combinations, not as a formal statistical estimate of generalization performance.

The variation in accuracy across the eight splits was significant, ranging from 0.678 to 0.944 with a standard deviation of 0.076. This indicates that some experimental combinations were substantially easier to classify than others. Such variation is informative in itself, demonstrating that experimental conditions do indeed influence spectral signals. Notably, even the lowest result (0.678) is well above random guessing (0.333), confirming the presence of a genuine biological signal. This variation also highlights that a single overall accuracy value should not be interpreted as a uniform performance level: the model performs well on some experimental combinations and poorly on others, and per-class and per-day analysis is necessary to understand where the errors occur. Still, we believe that cross-experimental validation should become the standard for research on hyperspectral plant diagnostics, as results lacking this validation are unlikely to be reproducible in the field.

The confusion matrix revealed a marked asymmetry: recall was 0.654 for stem rust, compared to 0.904 for net blotch and 0.850 for spot blotch. The primary confusion occurs between stem rust and spot blotch: approximately 25% of stem rust instances were misclassified as spot blotch, whereas only about 11% of spot blotch instances were misclassified as stem rust. The reason for this asymmetry remains unclear. A plausible but unverified factor is the difference in the duration of the asymptomatic period: under laboratory conditions, this period lasts 5–6 days for stem rust, compared to 2–3 days for the blotch pathogens [52,53,54]. However, our data do not allow us to establish a causal link between the duration of the asymptomatic period and classification difficulty, and other explanations cannot be excluded. Future studies should standardize the DAI scale relative to the onset of symptoms rather than the day of inoculation, thereby enabling a comparison of the pathogens under comparable physiological conditions.

Our work was based on a systematic ablation analysis: we compared three classifiers and four feature representations, yielding twelve combinations in total, and evaluated each across eight test splits. This approach enabled us to disentangle the contribution of the model from that of the feature representation [63,64,65,66]. The primary finding was unexpected: the choice of feature representation had a greater impact than the choice of classifier. The performance gap between the best and worst representations for a given model exceeded the difference between models using the same representation. This is methodologically significant, as the hyperspectral diagnostics literature often prioritizes algorithm comparison while devoting less attention to preprocessing and feature extraction, though some recent reviews do acknowledge the importance of this area [67,68,69]. Our results demonstrate that the treatment of the spectral curve’s shape is the key driver of accuracy improvements. We view ablation not as an end in itself, but as a tool to understand which spectral information is truly critical for pathogen differentiation. Future work could extend this approach by incorporating additional feature representations and evaluating them using the same data splits.

Among the three classifiers tested, PLS-DA yielded the best average results (0.725), followed by logistic regression (0.667), while kNN proved the least effective (0.512). The difference between PLS-DA and logistic regression falls within the range of observed variability (SD = 0.076), indicating comparable performance for both linear models. Notably, both consistently outperformed kNN across all feature representations. This aligns with the expectation that hyperspectral data are high-dimensional and exhibit strong inter-channel correlation [70,71,72]. PLS-DA constructs a small number of latent components that simultaneously account for feature variation and the relationship between features and classes, making it particularly well-suited for such data. Logistic regression with L2 regularization also handles multicollinearity effectively but does not explicitly leverage the data structure in the same way [47,48]. The k-nearest neighbors method appeared more sensitive to inter-experimental shifts, as evidenced by the high variance in kNN results across data splits. We view the choice of linear models as a deliberate decision based on the data structure, rather than a rejection of more complex methods.

Among the four feature representations, spline-based features yielded the highest average accuracy (0.803), followed by the first differences (0.788), the smoothed spectrum (0.756), and spectral indices (0.553). The difference between splines and the first derivative is small and falls within the range of standard deviation. However, both configurations consistently outperform the raw spectrum, and all three consistently outperform spectral indices. This leads to an important conclusion: the shape of the spectral curve, rather than absolute reflectance values, is the most informative factor for distinguishing between pathogens. Splines provide a compact, smooth description of the shape that is robust against high-frequency noise [73,74]. The first difference highlights local transitions but amplifies noise [74,75]. Spectral indices, being dimensionless combinations of spectral bands, lose shape information and thus prove to be the least informative, a finding supported by our earlier work and the existing literature [18,68,69,76]. This is consistent with the relatively poor performance of the 9 vegetation indices tested here (Table S1), which, despite their widespread use in plant stress detection, were unable to capture the subtle spectral differences between pathogens at early stages. These results align with our hypothesis that early-stage differences between pathogens manifest as subtle changes in the spectral profile rather than as variations in the overall reflectance level. This hypothesis emerged from our previous experiments, in which approaches based on absolute band values performed well on detached leaves but lost effectiveness when applied to intact potted plants [17,18]. Focusing on curve shape represents a promising avenue for future research into the early differentiation of diseases.

This study should be viewed as a proof-of-concept conducted under controlled laboratory conditions. All experiments were performed on cv. ‘Lyuboyar’ barley seedlings in a growth chamber where temperature, humidity, photoperiod, and light intensity were maintained at constant levels. While this design minimized external factors and allowed us to focus on spectral differences between pathogens, it also limits the applicability of the findings to real-world agronomic conditions. In the field, a leaf’s spectral signal depends on numerous additional factors not replicated in our experiments, such as plant age and physiological status, fluctuating light, and weather conditions [77,78,79]. Furthermore, single infections are rare in the field. Mixed infections typically occur, and the ratio of pathogens can shift depending on weather and plant developmental stages [80,81,82]. We used only a single cultivar, whereas different cultivars may exhibit distinct spectral responses even in the absence of a disease. Different isolates may also exhibit distinct spectral responses and this remains to be tested [83,84,85]. Finally, the model was trained on data from a narrow time window, and its robustness regarding seasonal and inter-annual variability remains untested. Consequently, deploying the model in the field will require additional validation and, most likely, further training using field data. A further limitation is the limited number of independent experiments per disease, namely two, which restricts the assessment of generalizability. While this is comparable to or exceeds the replication level of many hyperspectral plant pathology studies, broader validation across more experiments, cultivars, and environments will be required before the method can be considered robust. We regard the laboratory phase as a necessary initial step rather than a ready-made solution; field trials represent the next stage of our work. Finally, the classifier operates in a closed-set setting, where every sample must be assigned to one of the three known classes. An open-set formulation, in which the model could reject samples that do not match any known class, could provide a more informative picture of classification errors and is a promising direction for future work.

Despite the aforementioned limitations, our work provides one of the first quantitative benchmarks for the multi-class differentiation of three barley diseases at an early stage. The average balanced accuracy of 0.803 significantly exceeds random guessing (0.333) and rises above 0.900 starting from the third day post-inoculation. To the best of our knowledge, no previous studies have distinguished three pathogens sharing a similar early infection strategy using hyperspectral data and cross-experimental validation. This implies that the obtained values should be viewed as an initial benchmark for this task rather than an ultimate performance ceiling. We do not claim that an accuracy of 0.803 is sufficient for practical application in its current form. However, even this level of accuracy at an early stage can aid decision-making, particularly when used as a supplementary tool rather than the sole source of information. Our results demonstrate that the spectral signatures of the three pathogens differ sufficiently to be distinguished by machine learning methods at the early stage. However, the specific biochemical or physiological basis of these spectral differences remains unknown, and linking them to known compositional differences between the pathogens is not yet possible. In other pathosystems, fungal secondary metabolites such as beauvericin have been shown to differ between closely related species [86], suggesting that biochemical profiling could complement spectral approaches in future work. This paves the way for future research involving larger datasets, field conditions, a wider range of cultivars, and potentially more complex models. We hope our work will serve as a starting point for other researchers addressing this challenge.

## 5. Conclusions

This study demonstrates that hyperspectral imaging combined with machine learning can differentiate three barley pathogens at early stages of infection under controlled conditions, even when visual symptoms are nonspecific or absent. The systematic ablation revealed that the choice of spectral feature representation had a greater impact on performance than the choice of classifier, with spline-based features and PLS-DA yielding the most robust results. Stem rust was consistently more difficult to distinguish than the two blotch diseases. The reason for this remains unclear; a longer latent period is a plausible but unverified factor. Importantly, the results show that the spectral signatures of the three pathogens differ sufficiently to be distinguished by machine learning methods, although the specific biochemical or physiological basis of these spectral differences remains unknown. However, the obtained accuracy is not yet sufficient for direct field deployment. Field validation, multi-cultivar calibration, and testing under variable environmental conditions remain necessary before practical application. This work establishes a first benchmark for the multiclass differentiation of barley pathogens at early stages and provides a methodological framework for future studies.

## Data Availability

Data is available via the online download link https://drive.google.com/drive/folders/1FYlipKtT_kiYuJrWzaSKDcGnp9a42UUm?usp=sharing (accessed on 28 September 2026).

## Supplementary Materials

The following supporting information can be downloaded at: https://www.mdpi.com/article/10.3390/jof12100793/s1, Figure S1A. *Puccinia* *graminis* f. sp. *tritici* urediniospores under the microscope (40×). Figure S1B. *Pyrenophora teres* f. *teres* conidia under the microscope (40×). Figure S1C. *Bipolaris sorokiniana* conidia under the microscope (40×). Figure S1D. *Bipolaris sorokiniana* (spot blotch) symptoms development around 6-9 DAI. Figure S1E. *Pyrenophora teres* f. *teres* (net blotch) symptoms development around 6-9 DAI. Figure S1F. *Puccinia graminis* f. sp. *tritici* (stem rust) symptoms development around 7-11 DAI. Table S1. List of vegetation indices computed from the preprocessed spectra. Figure S2a. Mean spectral curves of the three barley disease classes. Raw spectra, averaged by class. Figure S2b. Mean spectral curves of the three barley disease classes. Raw spectra, averaged by experiment and class. Figure S2c. Mean spectral curves of the three barley disease classes after curve correction (cu), averaged by class. Figure S2d. Mean spectral curves of the three barley disease classes after curve correction (cu), averaged by experiment and class. Figure S2e. Mean spectral curves of the three barley disease classes after curve and channel correction (cu + ch), averaged by class. Figure S2f. Mean spectral curves of the three barley disease classes after curve and channel correction (cu + ch), averaged by experiment and class.

## Abbreviations

The following abbreviations are used in this manuscript:

ARI	Anthocyanin Reflectance Index
BBCH	Universal decimal scale for plant growth stages
CRI	Carotenoid Reflectance Index
DAI	Days After Inoculation
F1	F1 Score
GNDVI	Green Normalized Difference Vegetation Index
HSI	Hyperspectral Imaging
kNN	k-Nearest Neighbors
LogReg	Logistic Regression
MCARI	Modified Chlorophyll Absorption Ratio Index
NDRE	Normalized Difference Red Edge Index
NDVI	Normalized Difference Vegetation Index
NB	Net Blotch
PLS-DA	Partial Least Squares Discriminant Analysis
PRI	Photochemical Reflectance Index
PSRI	Plant Senescence Reflectance Index
SB	Spot Blotch
SD	Standard Deviation
SIPI	Structure-Insensitive Pigment Index
SR	Stem Rust
SVM	Support Vector Machine
TPE	Tree-structured Parzen Estimator
