# Supplementary material for: Early Differentiation of Barley Diseases (Net Blotch, Spot Blotch, and Stem Rust) Using Hyperspectral Imaging and Machine Learning
Source: J Fungi (Basel). 2026 Oct 21;12(10):793. doi: 10.3390/jof12100793 (PMC13706188; doi:10.3390/jof12100793)
Supplement: Supplementary file 1 [file jof-12-00793-s001.zip › Supplementary materials S1.pdf]

Supplementary materials S1.

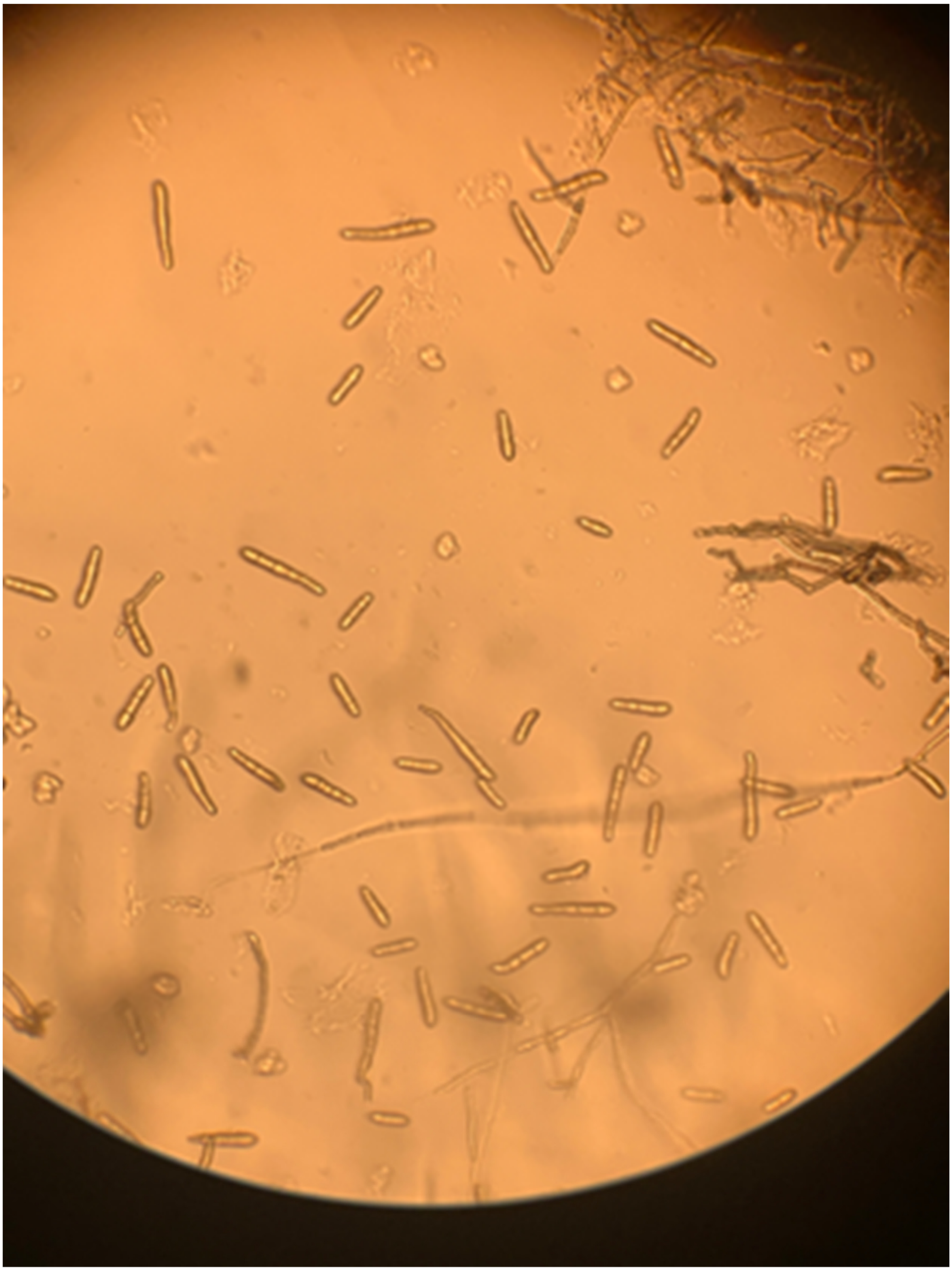

Figure S1A. *Pyrenophora teres* f. *teres* conidia under the microscope (40×).

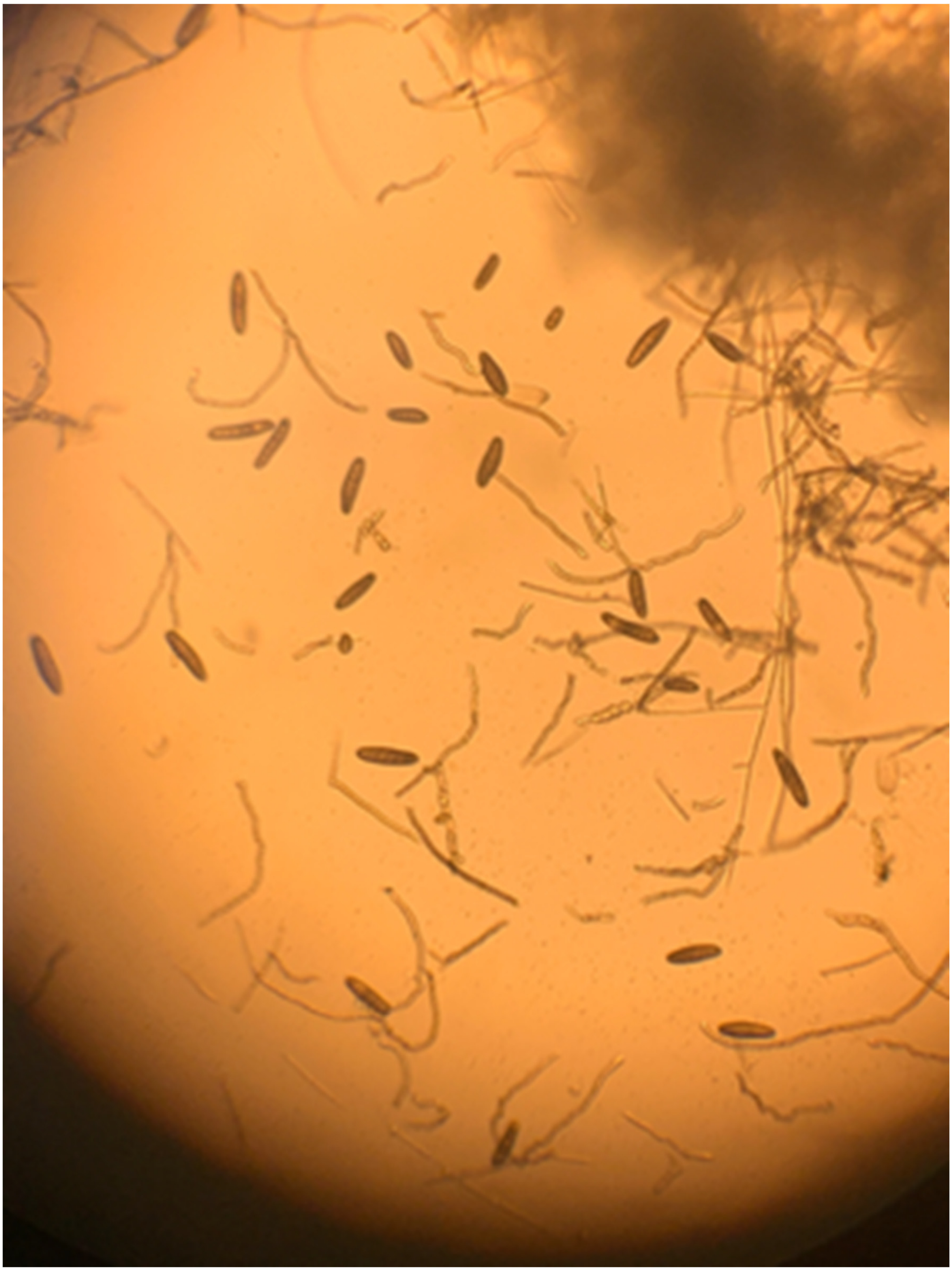

Figure S1B. *Bipolaris sorokiniana* conidia under the microscope (40×).

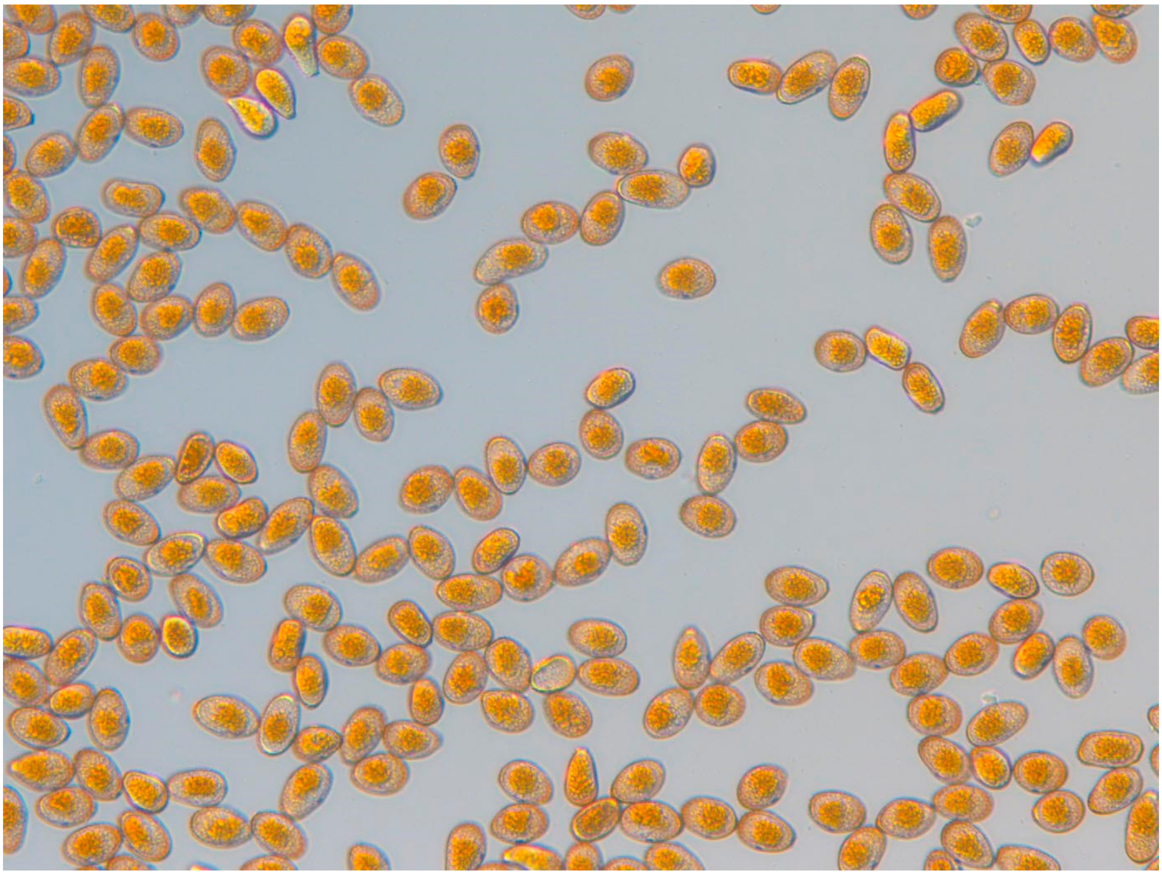

Figure S1C. *Puccinia graminis* f. sp. *tritici* urediniospores under the microscope (40×).

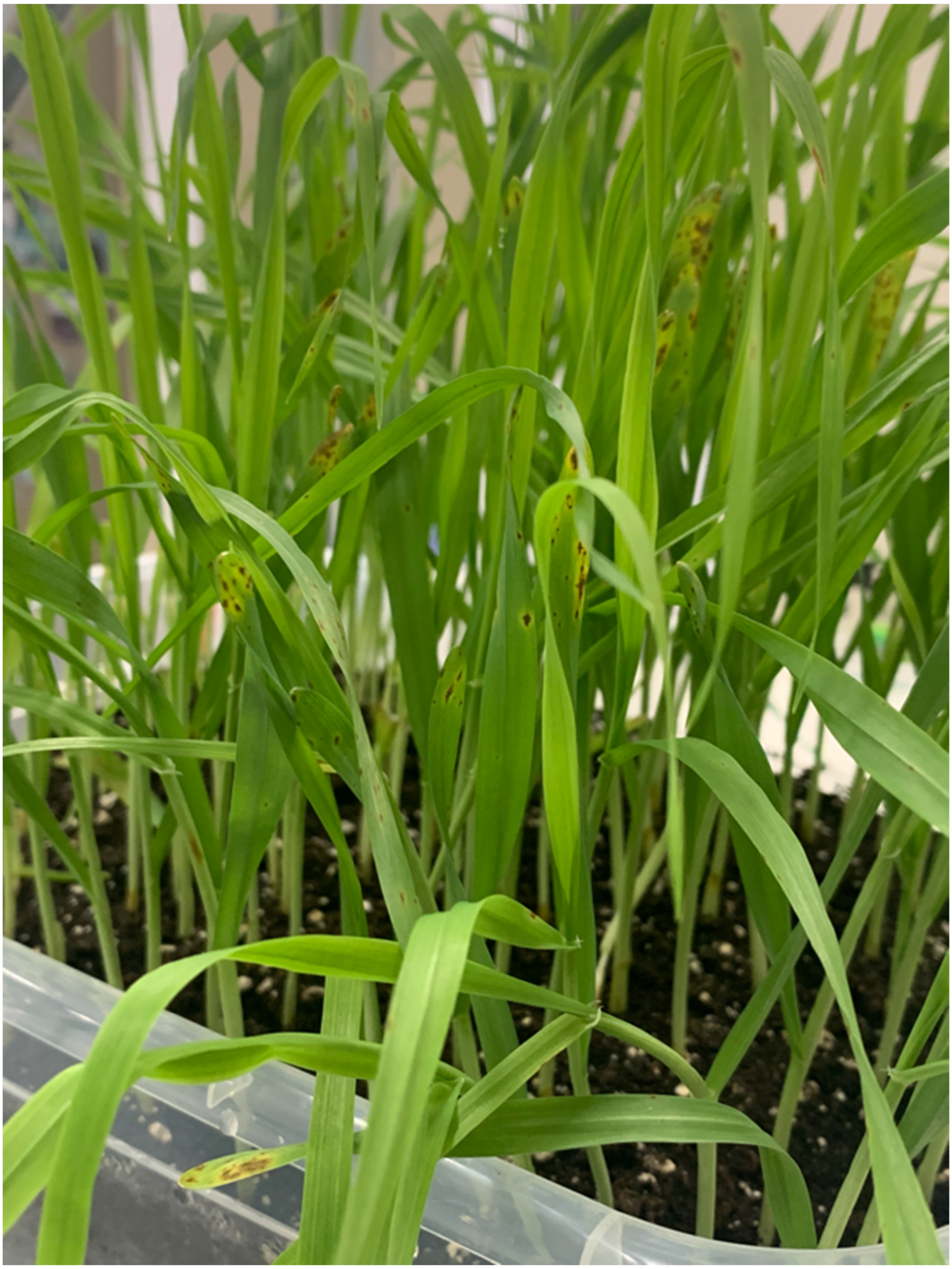

Figure S1D. *Bipolaris sorokiniana* (spot blotch) symptoms development around 6-9 DAI

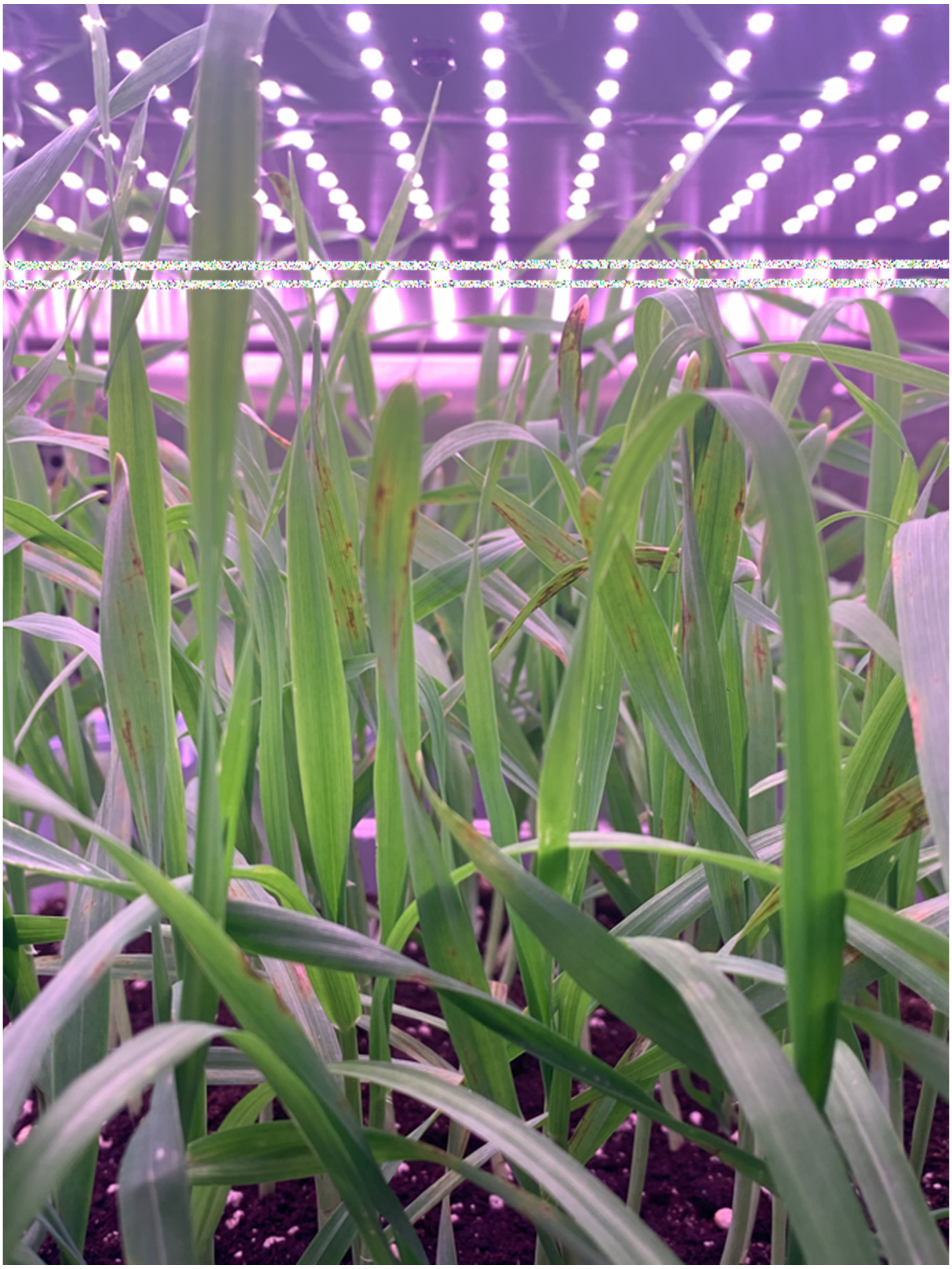

Figure S1E. *Pyrenophora teres* f. *teres* (net blotch) symptoms development around 6-9 DAI

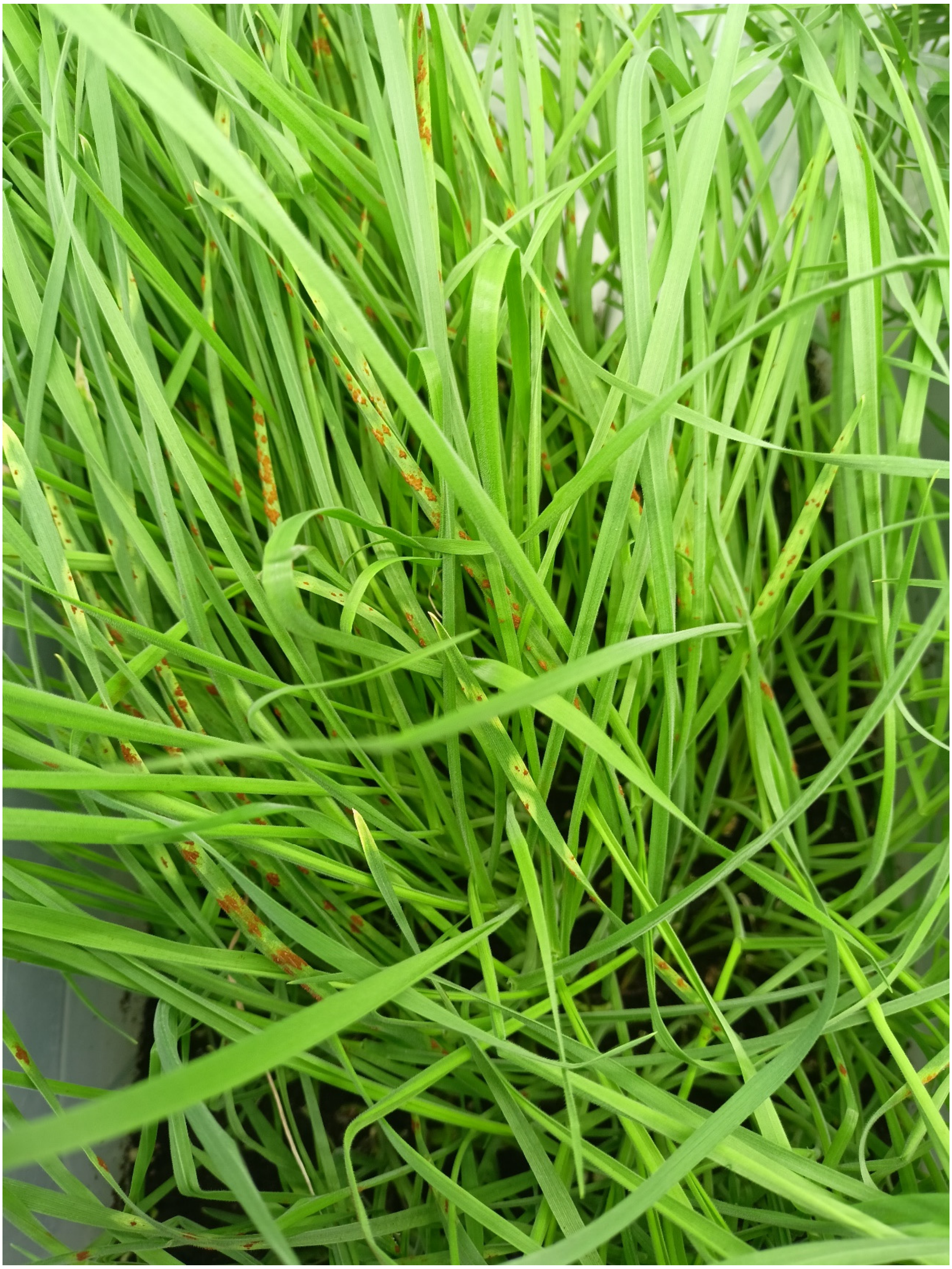

Figure S1F. *Puccinia graminis* f. sp. *tritici* (stem rust) symptoms development around 7-11 DAI
