# Supplementary material for: Early Differentiation of Barley Diseases (Net Blotch, Spot Blotch, and Stem Rust) Using Hyperspectral Imaging and Machine Learning
Source: J Fungi (Basel). 2026 Oct 21;12(10):793. doi: 10.3390/jof12100793 (PMC13706188; doi:10.3390/jof12100793)
Supplement: Supplementary file 1 [file jof-12-00793-s001.zip › Supplementary materials S2.pdf]

Supplementary materials S2

Table S1. List of vegetation indices computed from the preprocessed spectra.

Index	Full Name	Formula
**NDVI**	Normalized Difference Vegetation Index	$\frac{R802 - R670}{R802 + R670}$
**GNDVI**	Green Normalized Difference Vegetation Index	$\frac{R802 - R550}{R802 + R550}$
**NDRE**	Normalized Difference Red Edge Index	$\frac{R790 - R722}{R790 + R722}$
**PRI**	Photochemical Reflectance Index	$\frac{R530 - R570}{R530 + R570}$
**SIPI**	Structure Insensitive Pigment Index	$\frac{R802 - R450}{R802 - R682}$
**PSRI**	Plant Senescence Reflectance Index	$\frac{R682 - R502}{R750}$
**ARI**	Anthocyanin Reflectance Index	$\left(\frac{1}{R550}\right) - \left(\frac{1}{R702}\right)$
**CRI**	Carotenoid Reflectance Index	$\left(\frac{1}{R510}\right) - \left(\frac{1}{R550}\right)$
**MCARI**	Modified Chlorophyll Absorption Ratio Index	$[(R702 - R670) - 0.2 \times (R702 - R550)] \times \left(\frac{R702}{R670}\right)$
