# Supplementary material for: Early Differentiation of Barley Diseases (Net Blotch, Spot Blotch, and Stem Rust) Using Hyperspectral Imaging and Machine Learning
Source: J Fungi (Basel). 2026 Oct 21;12(10):793. doi: 10.3390/jof12100793 (PMC13706188; doi:10.3390/jof12100793)
Supplement: Supplementary file 1 [file jof-12-00793-s001.zip › Supplementary materials S3.pdf]

Supplementary materials S3

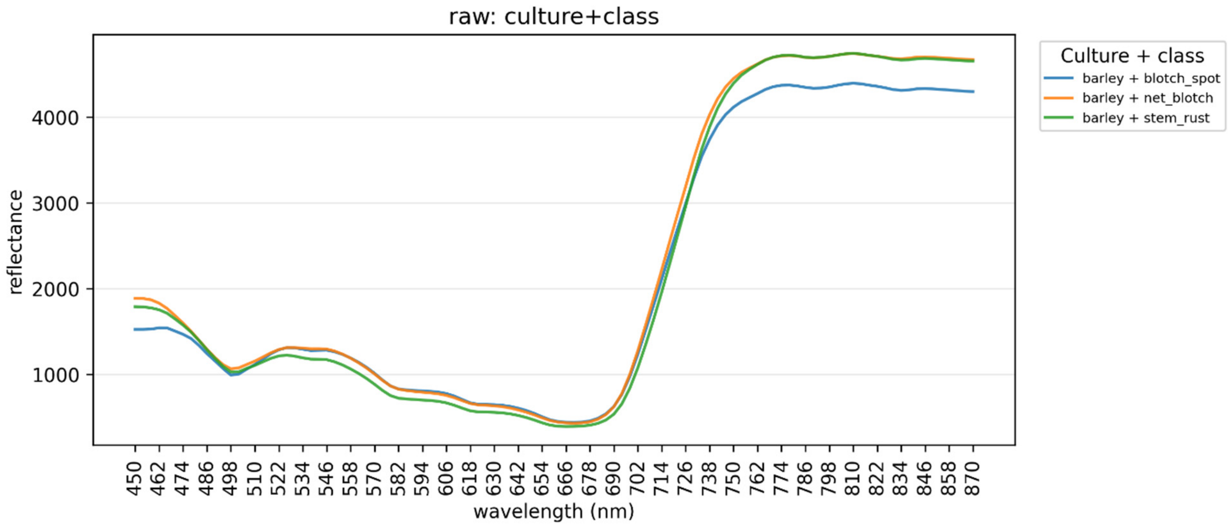

Figure S2a. Mean spectral curves of the three barley disease classes. Raw spectra, averaged by class.

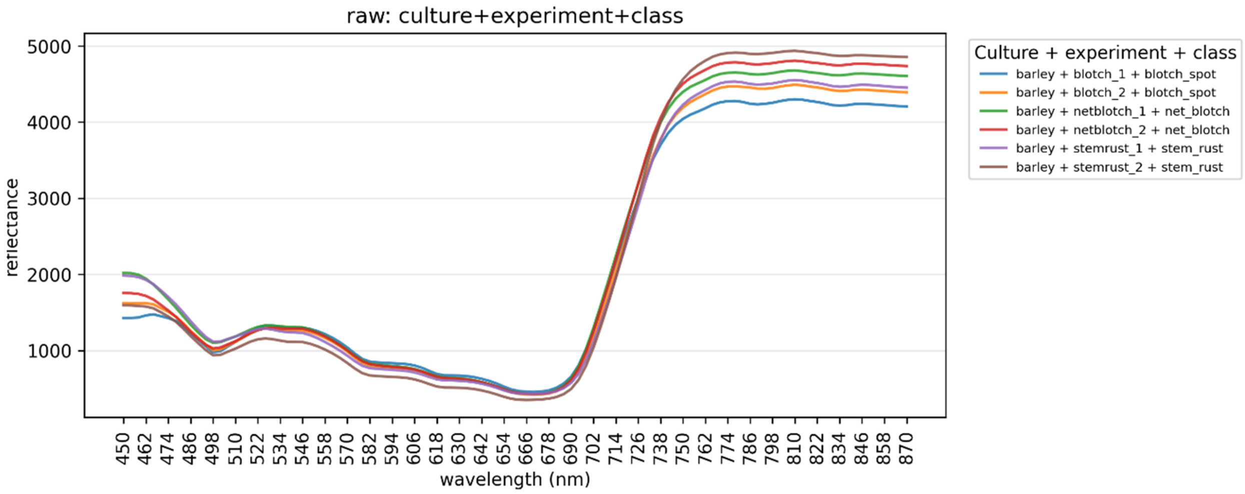

Figure S2b. Mean spectral curves of the three barley disease classes. Raw spectra, averaged by experiment and class.

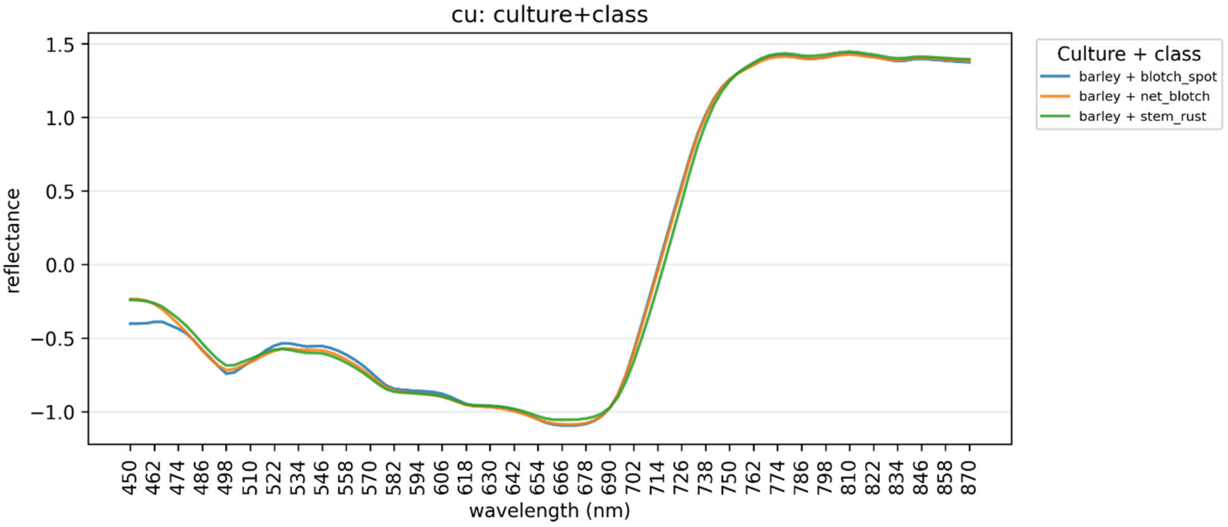

Figure S2c. Mean spectral curves of the three barley disease classes after curve correction (cu), averaged by class.

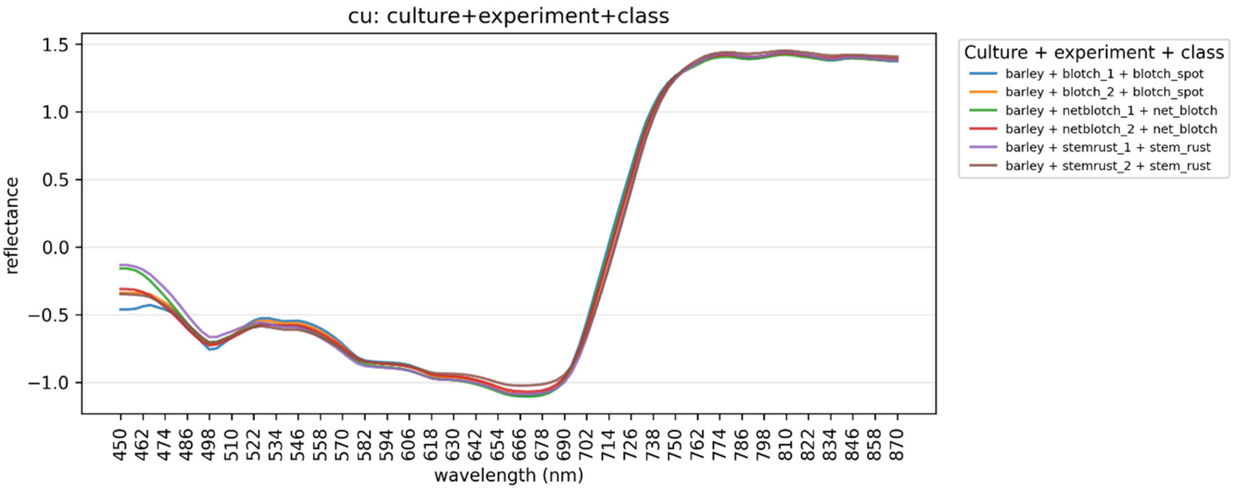

Figure S2d. Mean spectral curves of the three barley disease classes after curve correction (cu), averaged by experiment and class.

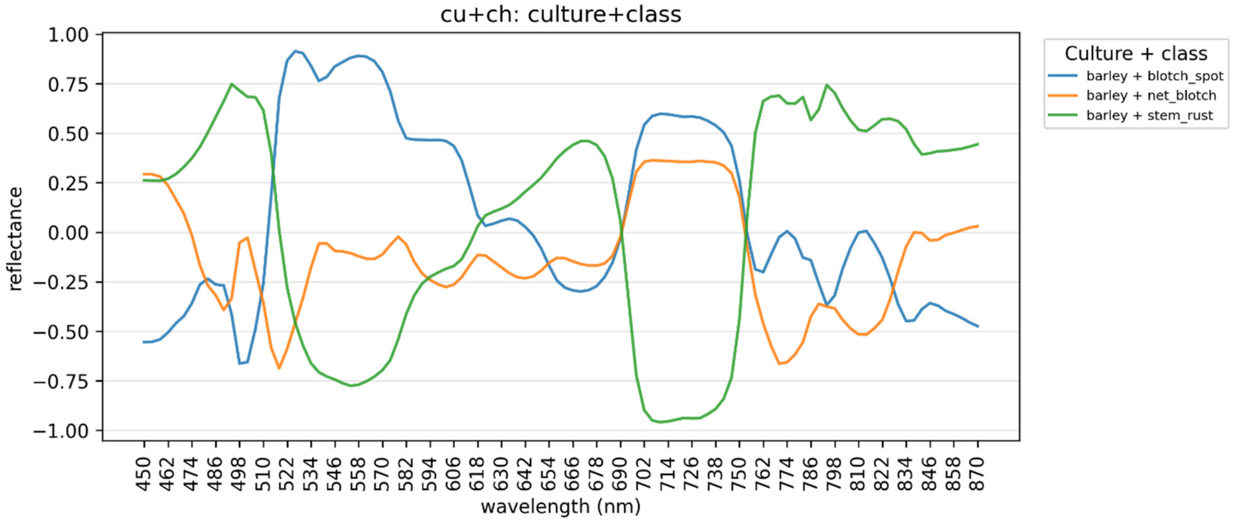

Figure S2e. Mean spectral curves of the three barley disease classes after curve and channel correction (cu + ch), averaged by class.

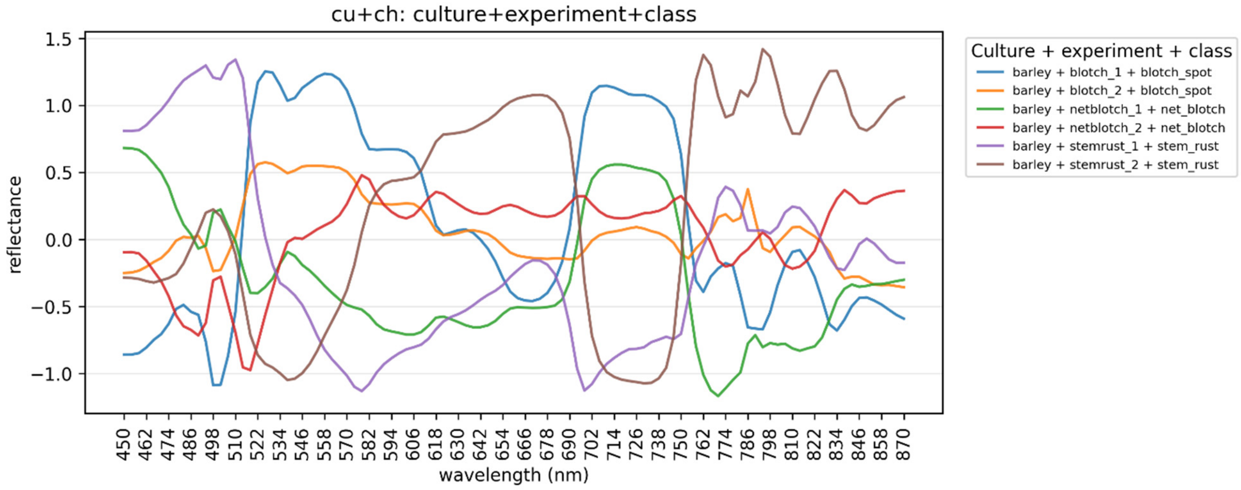

Figure S2f. Mean spectral curves of the three barley disease classes after curve and channel correction (cu + ch), averaged by experiment and class.
